# Dissipative Self-Assembly of Patchy Particles under Nonequilibrium Drive: A Computational Study

**DOI:** 10.1021/acs.jctc.4c00856

**Published:** 2024-10-04

**Authors:** Shubhadeep Nag, Gili Bisker

**Affiliations:** † Department of Biomedical Engineering, Faculty of Engineering, Tel Aviv University, Tel Aviv 69978, Israel; ‡ The Center for Physics and Chemistry of Living Systems, Tel Aviv University, Tel Aviv 6997801, Israel; § The Center for Nanoscience and Nanotechnology, Tel Aviv University, Tel Aviv 6997801, Israel; ∥ The Center for Light-Matter Interaction, Tel Aviv University, Tel Aviv 6997801, Israel; ⊥ The Center for Computational Molecular and Materials Science, Tel Aviv University, Tel Aviv 6997801, Israel

## Abstract

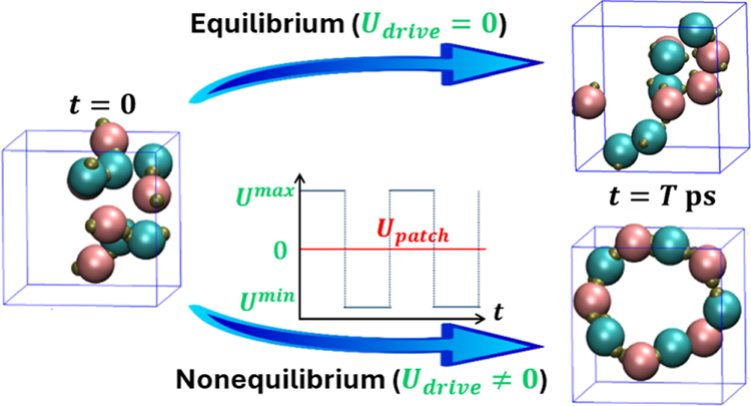

Inspired by biology and implemented using nanotechnology, the self-assembly of patchy particles has emerged as a pivotal mechanism for constructing complex structures that mimic natural systems with diverse functionalities. Here, we explore the dissipative self-assembly of patchy particles under nonequilibrium conditions, with the aim of overcoming the constraints imposed by equilibrium assembly. Utilizing extensive Monte Carlo (MC) and Molecular Dynamics (MD) simulations, we provide insight into the effects of external forces that mirror natural and chemical processes on the assembly rates and the stability of the resulting assemblies comprising 8, 10, and 13 patchy particles. Implemented by a favorable bond-promoting drive in MC or a pulsed square wave potential in MD, our simulations reveal the role these external drives play in accelerating assembly kinetics and enhancing structural stability, evidenced by a decrease in the time to first assembly and an increase in the duration the system remains in an assembled state. Through the analysis of an order parameter, entropy production, bond dynamics, and interparticle forces, we unravel the underlying mechanisms driving these advancements. We also validated our key findings by simulating a larger system of 100 patchy particles. Our comprehensive results not only shed light on the impact of external stimuli on self-assembly processes but also open a promising pathway for expanding the application by leveraging patchy particles for novel nanostructures.

## 1 Introduction

Self-assembly is a fundamental process in nature, in which particles within a bounded system spontaneously organize into an ordered structure, directed by intermolecular interactions. This phenomenon is not only pivotal in a myriad of biological processes, such as protein folding and cell membrane formation, but it also plays a crucial role in advancing nanotechnology, facilitating the creation of sophisticated structures across multiple scales.^1−7^ Particularly in nanoscience, self-assembly enables the development of innovative applications, ranging from drug delivery systems, exemplified by liposomes formed from lipid bilayers,^8−10^ to the construction of advanced materials for photonic and electronic sensing devices using organized gold nanoparticles.^11−13^ These diverse applications accentuate the importance of understanding and modeling self-assembly processes.

Self-assembly is categorized into two primary types: static and dissipative. In static self-assembly, the formed structure does not rely on any external source of energy to maintain itself, and the system minimizes its free energy under equilibrium conditions.^1^ Conversely, dissipative self-assembly occurs under nonequilibrium conditions, often at the expense of external energy sources, and the system dissipates energy to maintain its structure.^14−17^ This nonequilibrium behavior is driven by a continuous energy supply from various sources such as light,^18^ chemical fuels,^19,20^ electric,^21^ and magnetic fields,^22^ resulting in a nonzero difference between the forward and backward fluxes, thereby activating individual components and form the desired structures.

The theory of dissipative states, first introduced by Prigogine,^23^ is continuously developing and is found to be relevant to many biological phenomena.^15,24−28^ Particularly, the thermodynamics of continuous energy-driven structure forming systems in nonequilibrium conditions has been a rapidly developing field with the introduction of various design principles to model dissipative self-assembly.^29−35^ One major goal of these studies is to overcome the bottlenecks or trade-offs imposed by equilibrium or static self-assemblies.

Equilibrium self-assembly raises many unavoidable trade-offs, each reflecting the intricate balance systems must achieve. For example, a system might navigate a trade-off between kinetic and thermodynamic factors, such that it may become confined in kinetically trapped states, failing to achieve the most thermodynamically favorable configuration. This can result in suboptimal or inefficient assembly processes.^25^ Numerous design principles have been suggested to overcome kinetically trapped states.^36,37^ To circumvent these limitations, researchers have explored diverse self-assembly strategies, employing nonreciprocal interactions and driving systems from arrested dynamics toward lower-energy configurations.^38,39^ This strategy leverages nonequilibrium dynamics and programmable interactions, opening up new avenues for the creation of dynamic, self-organizing structures across different scales—from nucleic acids to colloidal particles, facilitating automated control over structural transitions in living systems.^40^ Additionally, Boekhoven and colleagues introduced a dissipative self-assembling system that utilizes external fuel, analyzing the associated entropy production and lost work.^41,42^ England explored the complex thermodynamics of nonequilibrium processes, crucial in understanding self-assembly and self-replication in biological systems.^28^ His work delves into the challenges of modeling nonequilibrium scenarios, especially in driven self-assembly, proposing mechanisms for self-organization via energy dissipation.^15,43,44^

Biological systems also exhibit these two types of assemblies. For instance, static self-assembly orchestrates the formation of functional hemoglobin proteins from hemoglobin polypeptides and functional ribosomes from the combination of RNA and ribosomal proteins.^45^ In contrast, dissipative self-assembly plays a crucial role in cell division and macromolecular assembly, such as tubulin dimer organization into microtubules facilitated by Guanosine triphosphate (GTP) hydrolysis, and chaperone-mediated macromolecule formation through Adenosine triphosphate (ATP) hydrolysis.^46−49^ Additionally, it is integral to active matter, driving self-organization in cellular cytoskeletons and bacterial colonies using external energy sources.^50^ Furthermore, experimental studies have highlighted the importance of ATP and GTP induced self-assembly systems,^51,52^ offering profound insights into the development of synthetic cells, prebiotic systems, and nanosystems.^53,54^ These advancements enhance our understanding of dissipative self-assembly modeling, crucial for deepening our comprehension of biological structures and offering significant potential in biochemical and materials sciences.^55−57^

The aforementioned example of the organization of tubulin dimers into microtubules, facilitated by GTP hydrolysis, serves as a primary example of dissipative self-assembly found in our cell.^47^ These microtubules feature a cylindrical shape with a hollow core, appearing as a ring of 13 dimers when viewed perpendicularly to the core’s radius.^58^ Similar ring-like self-assembly structures are found in circular DNA and cyclic protein complexes, which are crucial for understanding complex biological assembly processes.^59−61^ The significance of these rings extends into nanoscience, serving as foundational blueprints for developing self-assembled nanorings.^62,63^ These nanorings, far from being merely minuscule structures, are pivotal building blocks for transformative applications in drug delivery, nanoelectronics, and photonic devices.^64^ Therefore, modeling such ring-like structures, and utilizing the external sources of energy helps understand biological phenomena and design advanced materials.

Computational studies play a crucial role in advancing our understanding of structures created through dissipative self-assembly, offering deeper microscopic insights. Within this framework, patchy particles, characterized by distinct patches that enable selective, anisotropic binding, emerge as a fundamental model system.^65^ The theoretical foundation for these interactions traces back to seminal theories proposed by Boltzmann,^66^ and were first practically demonstrated with the synthesis of Janus particles, a notable subclass of patchy particles, in the late 1980s.^67^ The specific term “patchy particle” was later formally coined,^68^ and the introduction of this concept has unlocked vast possibilities in predicting crystal structures, thereby significantly contributing to the advancement of nanoscience and nanofabrication. This breakthrough has sparked a wave of research, exploring the broad applications and potential of patchy particles in various domains.^69−74^ For example, Gong et al. synthesized patchy particles using colloidal fusion.^71^ Additional study demonstrated the synthesis of Janus particles using evaporative deposition of preferential materials on spheroid, changing the properties of one hemispheroid.^75^ Chang and others showed a simple yet effective technique by using seed-mediated heterogeneous nucleation to synthesize patchy particles.^76^ These patchy particles have been found useful in many potential applications such as targeted drug delivery,^77,78^ photonic crystals,^79−82^ phoretic motors,^83^ and more. Recent explorations into the self-assembly of patchy particles under external stimuli, such as electric^84^ and magnetic fields,^85^ have further highlighted their role in advancing new material designs.

In computational research, patchy particles have been extensively employed to simulate diverse systems, such as protein droplets^86,87^ and colloidal assemblies,^65^ enabling a profound exploration of phenomena like crystal nucleation and growth,^88,89^ liquid–liquid phase transitions,^86^ and protein folding,^90^ among others.^91−93^ By tuning the characteristics of patches, models based on patchy particles have offered insights into the phase behavior of colloids.^94^ Specifically, Nguemaha et al. utilized Monte Carlo (MC) simulations with two-component patchy particles to investigate RNA’s role in the liquid–liquid phase separation observed within membrane-less organelles, focusing on protein mixtures that incorporate regulatory components.^86^ Similarly, Gnan, Sciortino, and Zaccarelli’s coarse-grain modeling of proteins using patchy particles has shed light on protein phase behavior.^87^ Espinosa et al. have also contributed by employing Molecular Dynamics (MD) simulations with patchy particles to demonstrate deviations from the law of rectilinear diameter through an examination of liquid–vapor coexistence phase behavior.^95^ Thus, the well-established understanding of patchy particles positions them as an ideal model system, which aims to leverage these insights further to understand biological self-assembly phenomena and pioneer new materials.

Designing such new materials requires the mitigation of the aforementioned trade-offs effectively. One unique approach to achieve this is by driving the system into nonequilibrium conditions, for example, through the introduction of a self-healing driving force.^96^ This proposed model of dissipative self-assembly is based on information from equilibrium self-assembly and biological processes such as the use of chemical fuel to organize structures with specific functions similar to the way microtubules are formed by α-tubulin and β-tubulin through GTPase activity.^97^ Inspired by these processes, it was demonstrated that nonequilibrium driving enhances dimer-based self-assembly, enabling smaller critical seeds and improved stability of target structures compared to equilibrium scenarios.^98^ Using a similar model, the stochastic landscape method was proposed for improving the predictive power for first assembly times, offering a quantitative framework for understanding and controlling nonequilibrium self-assembly processes.^99^ Recently, it has been used to classify protein states.^100^

In the present study, we aim to expand this approach of nonequilibrium driving force by undertaking a detailed exploration of self-assembly mechanisms using patchy particles in three-dimension (3D), employing a comprehensive simulation approach. Our investigation begins with equilibrium MC simulations across three distinct systems, each comprising 8, 10, and 13 patchy particles, respectively, where each particle has two possible internal states. These particles are specifically designed to achieve target structures of *n*-sided polygons or *n*-ring configurations, comprising all the particles in the system. We examine the formation dynamics and stability of the self-assembled structures, particularly in relation to the varying interaction energies between patches, and identify a domain of equilibrium trade-offs, delineating a complex relationship between assembly time and structural stability. To overcome these trade-offs, an external bias is introduced, shifting the dynamics to the nonequilibrium regime, inspired by biological systems that exploit energy molecules like ATP and GTP thus mimicking the continuous energy supply essential for sustaining assembly processes in living systems.^101^ A rigorous thermodynamic analysis encompassing entropy production and total energy computations shows how this externally applied drive enables the system to form the target structure faster compared to the equilibrium case. Complementing the MC results, we perform MD simulations for systems of 8 and 10 patchy particles under both equilibrium and nonequilibrium conditions. For nonequilibrium MD simulations, a square wave potential is applied periodically as an external drive, where we further analyze the bond formation and breakage statistics, the underlying forces, and an order parameter to distill the underlying mechanism governing the dissipative self-assembly. We demonstrate how the oscillating potential induces momentary forces that accelerate the assembly, allowing the system to rapidly achieve the target structure. By revealing the nuanced dynamics of dissipative self-assembly with patchy particles, this study highlights the critical role of external energy in guiding assembly processes. Our insights offer a foundation for novel approaches in biophysics and material engineering, setting the stage for advancements in the design and control of self-assembling systems.

## 2 Computational Framework

### 2.1 Simulation Cells

**Figure 1:**
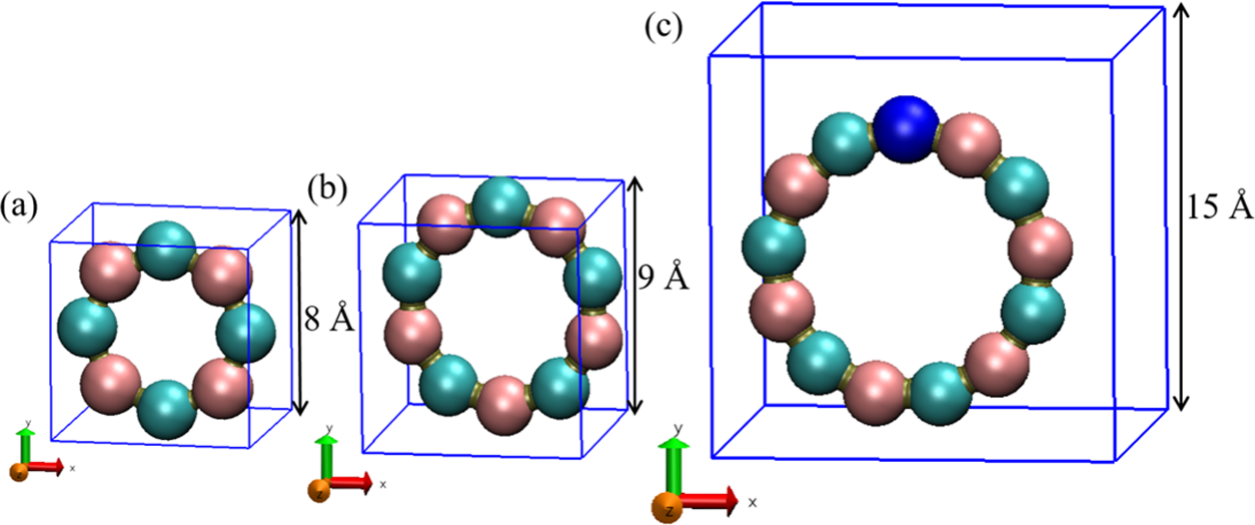
Target structures of (a) 8-patchy particle system, (b) 10-patchy particle system, and (c) 13-patchy particle system. Each target structure is presented within its cubic simulation cell, with lattice parameters set to 8, 9, and 15 Å for the 8, 10, and 13-particle systems. In the 8 and 10-particle systems, particles in states α and β are represented by pink and cyan colors, respectively. For the 13-patchy particle system, the γ state appears in blue.

MC simulations were conducted for three systems, comprising 8, 10, and 13 patchy particles, whereas MD simulations were conducted for systems with 8 and 10 patchy particles. Each particle was designed with two surface patches and varied states depending on the system. In the systems with 8 and 10 patchy particles, we divided the particles into two distinct states, labeled α and β, with equal distribution among the particle populations. Conversely, in the system comprising 13 patchy particles, we introduced a third state, γ, alongside the existing α and β states. In this scenario, six particles were assigned to the α state and another six to the β state, with the remaining particle embodying the γ state. Our objective was to form target structures that emulate polygons with 8, 10, and 13 sides, corresponding to each system (Figure 1). To realize these structures, the patches on each particle were meticulously positioned to ensure that the angle between two lines—each extending from a patch to the particle’s center—matched the interior angles of an 8-sided, 10-sided, and 13-sided polygon, respective to each system. These angles are 2.356, 2.513, and 2.658 rad for 8, 10, and 13 patchy particle system, respectively. Furthermore, the size of the cubic simulation boxes was carefully chosen based on the number of patchy particles to be 8, 9, and 15 Å for the 8-particle, 10-particle, and 13-particle systems, respectively. In MD simulation, we simulated 8 and 10 particle systems with the same simulation parameters as described above, except that the particles had only one internal state, α.

### 2.2 Interaction Potential of Patchy Particles

Patchy particle simulations have been a versatile tool for modeling a wide range of structures, ranging from proteins and DNA to small molecules and atoms.^102^ Kern and Frenkel proposed an approach for simulating patchy particles by modeling the bead of the patchy particles with hard-sphere potential and patches with both square well potential along with an angular dependent potential.^103^ This model has been shown effective in simulating many cases of patchy particles.^88,104−107^

**Figure 2:**
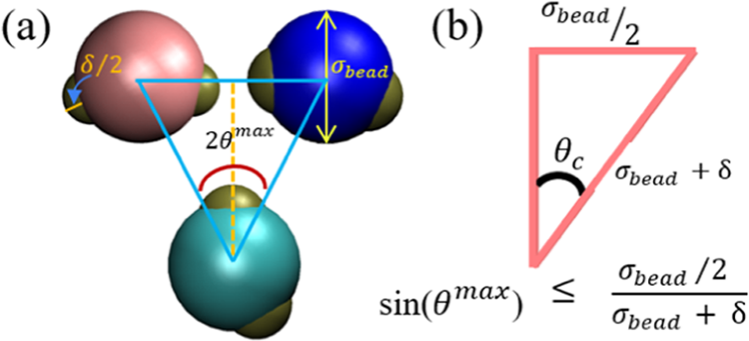
Patchy particles model. (a) A schematic where three patchy particles may potentially interact with each other, resulting in an unwanted case of multiple bonds per patch. (b) A specific condition is implemented to prevent more than a single bond per patch (see details in the main text). This figure is a schematic representation created to visualize the concepts described, with dimensions and proportions aligned with those used in our simulations.

We build upon the Kern–Frenkel approach and apply it to our system of 8, 10, and 13 patchy particles. Based on this approach, each patchy particle is featured with patches of an angular width of 2θ^max^ centered to the core of the bead and length δ/2 (Figure 2a). In this framework, the total interaction energy of a patchy particle, *U*_Particle_, is
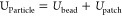
1where *U*_bead_ and *U*_patch_ are the energies associated with the bead and the patch, respectively.

The expression of *U*_bead_ is modeled as Lennard-Jones (LJ) interaction:
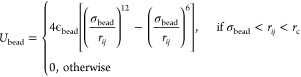
2where ϵ_bead_ is the depth of the potential well of the bead and σ_bead_ is its van der Waals (vdW) radius. These two values are set at 0.75 kJ/mol and 2 Å throughout the simulation. When the distance between two patchy particles *i* and *j*, denoted as *r*_*ij*_, is smaller than the cutoff distance, *r*_c_, the particles interact. Here, *r*_c_ is taken as 5 Å.

In the case of MC simulation of 8 and 10 patchy particles, the two different states α and β attract each other, whereas the same states do not interact with each other (*U*_patch_^αα^ = 0, *U*_patch_^ββ^ = 0). The patch energy *U*_patch_^αβ^ between α and β states of the 8 and 10 patchy particle systems is
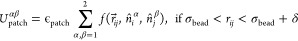
3where ϵ_patch_ denotes the depth of the potential between patches of the particle, *r⃗*_*ij*_ is the vector connecting the center of particle *i*^th^ to the center of particle *j*^th^, *n̂*_*j*_^α^ or *n̂*_*j*_^β^ denote the unit vector connecting the center of the bead and the center of patches of the patchy particle of state α or β, respectively, and *f*(*r⃗*_*ij*_, *n̂*_*i*_^α^, *n̂*_*i*_^β^) is the angular dependence in the interaction between patches, and which is given by
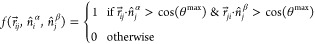
4This condition ensures that the patchy interaction potential is activated only when the angle between *r⃗*_*ij*_, and the unit vectors *n̂*_*i*_^α^ and *n̂*_*j*_^β^, representing the orientation of their respective patches, falls within half of the angular width of the patch, θ^max^. Specifically, this occurs when both *r⃗*_*ij*_ · *n̂*_*i*_^α^ and *r⃗*_*ij*_ · *n̂*_*j*_^β^ are larger than the cosine of θ^max^. When these conditions are satisfied, meaning the patches are correctly aligned, the angular dependency term equals 1, and the interaction potential *U*_patch_ of eq 1 contributes to the total interaction energy of the patchy particle.

For the 13 patchy particles system, there are three internal states: α, β, and γ. The α and β states interact with each other similar to the 8 and 10 particle cases. The interaction between the γ state and the α or β states is the energy *U*_patch_^α/βγ^, with a similar form as eq 3 and the corresponding angular dependence *f*(*r⃗_ij_*, *n̂*_*i*_^α/β^, *n̂*_*j*_^γ^) similar to eq 4. This ensures that the interaction potential *U*_patch_ is activated only when both the angle between *r⃗*_*ij*_ and the unit vector *n̂*_*i*_^α/β^, and the angle between *r⃗*_*ji*_ and *n̂*_*j*_^γ^, fall within half of the angular width of the patch, θ^max^. Here, *n̂*_*j*_^γ^ denotes the unit vector connecting the center of the bead and the center of patches of the γ patchy particle.

To ensure a single bond per patch,^102,108^ the length of the patch must be related to the angular width by the inequality sin(θ^max^) ≤ (σ_bead_/2)/(σ_bead_ + δ). To satisfy this condition, we chose θ^max^ to be 0.35 radians. The schematic of this condition is shown in Figure 2b.

### 2.3 Initial Conditions

To rigorously assess the inherent trade-offs associated with equilibrium simulations and to determine how an external bias can mitigate these limitations, our investigation centers on two critical metrics, namely, the time until the first self-assembly event, *T*_fas_, and time the system remains in the assembled state as a proxy for target stability, *T*_stable_. Accordingly, our approach in both MC and MD simulations encompassed two distinct methodologies in which the initial conditions were set to be random, for analyzing *T*_fas_, or as the target structure, for analyzing *T*_stable_. In the former, the simulation was initiated with patchy particles distributed randomly in the simulation box, designed to meticulously observe and record the time frame leading to the first emergence of the target structure. In the latter case, the simulation was initiated with a preformed target structure to diligently monitor and analyze the enduring stability and structural integrity of the assembly. Using these two different initial conditions, we aimed to acquire a comprehensive understanding of the dynamics and robustness of the structures formed under equilibrium and nonequilibrium conditions. Throughout our study, we presented the median values of *T*_fas_ and *T*_stable_, and showed the full distribution of the assembly and disassembly times whenever possible. The rationale is attributed to the fact that in order to compute the mean, we must consider realizations in which the systems successfully assemble for *T*_fas_ or disassemble for *T*_stable_. These would be highly computationally expensive, particularly for lower interaction energies and very high interaction energies, respectively.

### 2.4 Monte Carlo Simulation

Our in-house program of patchy particles included both equilibrium and nonequilibrium MC simulations. The MC moves comprised both the translation of the beads and the rotation of patches relative to the center of the bead. All the MC simulations in this work were performed at a temperature of 65 K, and we have employed reflective walls. The simulation lengths, in terms of the number of MC steps, were set to 10 × 10^6^ for realizations starting from both random and target configurations to determine *T*_fas_ and *T*_stable_ for the 8 and 10 patchy particle systems. For the 13-patchy particle system, given its increased complexity and the intricate nature of the target structure, the number of MC steps was increased to 15 × 10^6^ to adequately capture the assembly process and ensure the formation of the target structure.

#### 2.4.1 Equilibrium Simulations

In equilibrium MC simulation, each step involved assessing the total interaction energy of the particles, both before and after a proposed MC step. We then employed the Metropolis criterion,^109^ which provided the probability for accepting or rejecting a move, according to the energy difference, min[1, *e*x*p*{−(*U*_Particle_^new^ – *U*_Particle_^old^)/*k*_B_*T*}], where *U*_Particle_^new^ and *U*_Particle_^old^ represent the new and old total energies, *k*_B_ is the Boltzmann constant and *T* is the temperature.

#### 2.4.2 Nonequilibrium Simulations

In our nonequilibrium MC simulation, we introduced an external driving force, denoted as ϵ_drive_, to the patches of the particles. This external force disrupted the equilibrium state of the system by deviating from the detailed balance condition, significantly changing the interaction dynamics between the particles. We applied this bias in a targeted manner, depending on whether the patches were moving toward or away from each other, aligning with the topology of the desired target structure to both form and maintain it.

The acceptance or rejection of a proposed move was adjected to include the drive. If two patches of two different patchy particles, which were the nearest neighbor of each other in the target structure, came into proximity in an MC step, the drive would render the proposed move more favorable by modifying the acceptance probability to min[1, exp{−(*U*_Particle_^new^ – ϵ_drive_ – *U*_Particle_^old^)/*k*_B_*T*}]. When two neighboring patches, which are adjacent in the target structure, move apart during an MC step, the drive would render the bond breaking less probable, by the modified acceptance probability to min[1, *exp*{−(*U*_Particle_^new^ + ϵ_drive_ – *U*_Particle_^old^)/*k*_B_*T*}]. When a virtual bond between two nearest neighbor patches, according to the target structure, persisted over successive MC steps, no external bias was added nor subtracted, and the acceptance probability followed the traditional Metropolis algorithm. The application of an external driving force (ϵ_drive_) in nonequilibrium simulations is hypothesized to significantly enhance the efficiency of target structure formation and its subsequent stability, by selectively favoring assembly pathways that align with the desired target structure.

Our approach of external drive replicates the directed self-assembly processes found in experimental systems like DNA nanostructures and colloidal crystals.^110−113^ In these experiments, specific interactions and external stimuli, such as light, pH variations, and electromagnetic fields, guide the formation of the desired structures. For instance, light and pH control in DNA assembly and disassembly enables DNA structures to adapt to various stimuli, exhibiting properties necessary for synthetic cells.^110−112^ Similarly, applying magnetic fields in colloidal crystal assembly selectively stabilizes favorable configurations by adjusting energy levels.^113^ These experimental analogs ensure that our simulations are grounded in realistic and practically achievable conditions.

Complementary to the 8, 10, and 13 patchy particle simulations aimed to understand the formation and stability of the target structures, comparative analyses were conducted to assess the effects of patch length variation and simulation cell dimension variation. These were carried out in 10 patchy particle systems to achieve insights into the factors influencing the self-assembly.

### 2.5 Molecular Dynamics Simulations

#### 2.5.1 Equilibrium Simulations

Equilibrium MD simulations were conducted using the Large-scale Atomic/Molecular Massively Parallel Simulator (LAMMPS) software^114^ within the canonical ensemble. We focused on two specific systems comprising 8 and 10-patchy particles, each featuring only a single internal state. Consistent with the MC simulations, the dimensions of the simulation cell for these systems were maintained at 8 and 9 Å, respectively, and periodic boundary conditions (PBC) were employed in all directions. The target structure for these simulations, depicted in Figure 1a and b, maintained uniform colors for all beads across both systems according to the single state particles.

Following the study of Jover et al., the bead of the patchy particle is modeled with pseudohard-sphere (PHS) potential:^115^
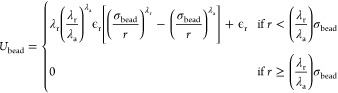
5where λ_a_ = 49 and λ_r_ = 50 are the coefficients for attractive and repulsive forces, respectively. The parameter ϵ_r_ represents the interaction strength in the PHS model, characterizing the energy associated with the interactions. The variable *r* signifies the distance measured from the center of one particle to another. Further, the values for ϵ_r_ and σ_bead_ are set to be 0.24 kJ/mol and 2 Å, respectively, aligned with the parameters utilized by Espinosa et al. in their investigation of liquid–vapor coexistence in systems comprising patchy particles.^95^ These parameters have been established to provide a realistic representation of particle interactions within the simulated environment.

The interaction energy between patches of different particles, *U*_patch_^csw^, is modeled with a continuous attractive square-well potential:^116^
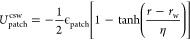
6where *r* refers to the distance between the centers of two patches on different particles, *r*_w_ is the radius of the attractive well, while the steepness of the well is controlled by η. In this context, *r*_w_ is equivalent to the width of the patches previously introduced in the simulation. In MD simulations, we have chosen the masses of the interaction sites to be 10% of the mass of the bead particle. The specific values of the core particle and patch were taken as 10 and 1 amu. However, the choice of masses might affect the quantitative nature but does not affect the qualitative nature of the key findings of our study since our external drive protocol does not depend on inertia. To ensure that only one bond forms per patch, we have set the values of η and *r*_w_ to be 0.005σ_bead_ and 0.12σ_bead_ respectively.

MD simulations were performed at a temperature of 40 K using the Nosé–Hoover thermostat,^117,118^ which is lower compared to the 65 K set in MC, to minimize fluctuations in the system. Nevertheless, employing two different temperatures does not affect our conclusions, as the system dynamics inherently depend on the ratio of ϵ_patch_ to *k*_B_*T*. The simulation duration for studying the self-assembly formation from both random initial conditions and starting at the target configuration was set to 12 000 ps. We integrated the equation of motion using the velocity Verlet algorithm^119^ with a carefully selected time step of 0.2 fs, chosen to ensure the stability and nondivergence of the simulations. During these simulations, we recorded the trajectories and total interaction energy between patches every 100 fs.

#### 2.5.2 Nonequilibrium Simulations

In our nonequilibrium MD simulations using LAMMPS, we have integrated an external drive of a square wave potential *U*_square_(*t*). Drawing inspiration from various biological systems, such a drive resembles the voltage-gated ion channels found in neurons that open and close in response to voltage changes,^120^ allowing for quick transmission of electrical signals. It is also similar to the bistable genetic switches that enable cellular phase transitions under specific conditions.^121^ Recent experimental studies, such as the one conducted by Song et al.^84^ and the capability of electromagnetic fields to direct the assembly of nonspherical particles studied by Shields et al.,^85^ further support our approach.

To simulate the influence of an external periodic drive, a square wave potential, *U*_square_(*t*), is employed to modulate patchy particle interactions within the LAMMPS framework,^114^ defined as
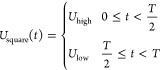
7where *U*_high_ and *U*_low_ correspond to the high energy and low energy amplitudes, and *T* is the period of the oscillation. This approach simulates the effect of an external drive by periodically shifting the interaction energy between the patchy particles, thus driving the system away from equilibrium.

By adding the square wave potential to the base patchy interaction potential, *U*_patch_^csw^ in eq 6, we introduce a time-dependent interaction potential that oscillates between two defined energy states. These states, corresponding to the high and low energy phases of the square wave, simulate the presence of an external driving force acting on the particles (see eq 8 below). This method provides a computationally feasible technique to study the dynamic self-assembly processes under nonequilibrium conditions.

The interaction potential between two patchy particles now includes two types of terms, a spatial-dependent term, *U*_patch_^csw^(*r*), and a time-dependent term, *U*_square_(*t*) (see eq 7). The new modified interaction potential, *U*_patch_^mod^(*r*, *t*), is given by
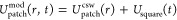
8and the modified total interaction energy of a patchy particle is
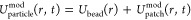
9where *U*_patch_^mod^(*r*, *t*) oscillates around the intrinsic patchy interaction potential, *U*_patch_^csw^(*r*). This oscillation introduces nonequilibrium conditions by periodically altering the interaction landscape.

**Figure 3:**
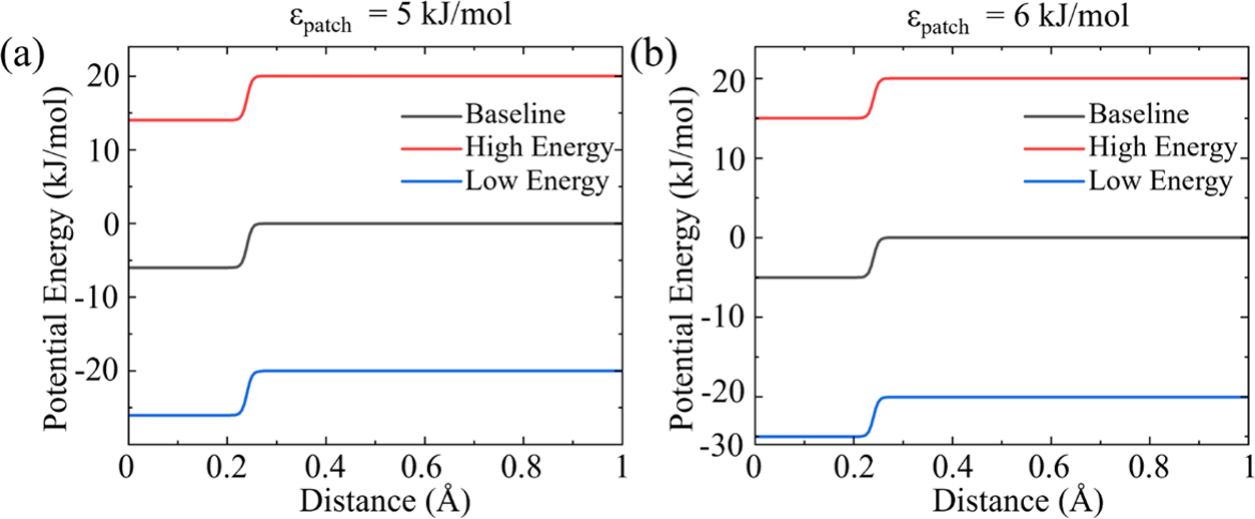
Patchy interaction potential, ϵ_patch_, having a baseline value (black) of (a) 5 kJ/mol and (b) 6 kJ/mol, and the corresponding high energy (red) and low energy (blue) phases of the square wave potential with a potential of 20 kJ/mol.

Figure 3 provides a visual representation of the square wave potential modulation of the interaction potential between patchy particles for baseline interaction energy of 5 kJ/mol (Figure 3a), and 6 kJ/mol (Figure 3b), with their respective high and low energy phases and an external wave potential amplitude of 20 kJ/mol.

The period of the square wave, set to 20 ps, ensures a rapid effect relative to the bond formation time scale, with amplitude variations directly mimicking the external drive adjustments in the nonequilibrium MC simulations. These settings allow us to explore the dynamics of self-assembly and structural stability under nonequilibrium conditions through *T*_fas_ and *T*_stable_. Excluding the periodic square potential, the rest of the simulation details, including the time-step, frequency of storing trajectories, and boundary conditions, were similar to the case of the MD simulation under equilibrium conditions.

In both MC and MD simulation, all the values of *T*_fas_ and *T*_stable_ were obtained from 20 distinct simulations for a given ϵ_patch_ and ϵ_drive_ values. Randomization of the 20 MC simulations stems from the different values of the random seed generator, whereas for MD simulations, randomization stems from different velocity distributions from the random seed generator.

We also simulate both MC and MD for a larger system comprising 100 particles in a simulation box with dimensions of 30 × 30 × 30 Å^3^, maintaining a number density of 0.003 Å^–3^. This is consistent with our previous simulation of 13 patchy particles in a cubic box with a side length of 15 Å, using a single interaction state between the patches. The simulation cell walls are modeled with PBC. The duration of the MC simulations is 2 × 10^6^ MC steps, and the MD simulation is 10 ns. During the simulation, we maintain a neighbor list with a cutoff distance of 10 Å, updating this list every 10 MC steps for MC simulation and 2 fs for MD simulation. The particles interact with each other through PHS potential (eq 5), and patches are modeled with angles set to match an 8-ring structure and a 4-ring structure while keeping other parameters identical to those used in the smaller-scale systems. For this large system, both MC and MD simulations were carried out at 40 K.

## 3 Results and Discussion

### 3.1 Monte Carlo Simulation Results

**Figure 4:**
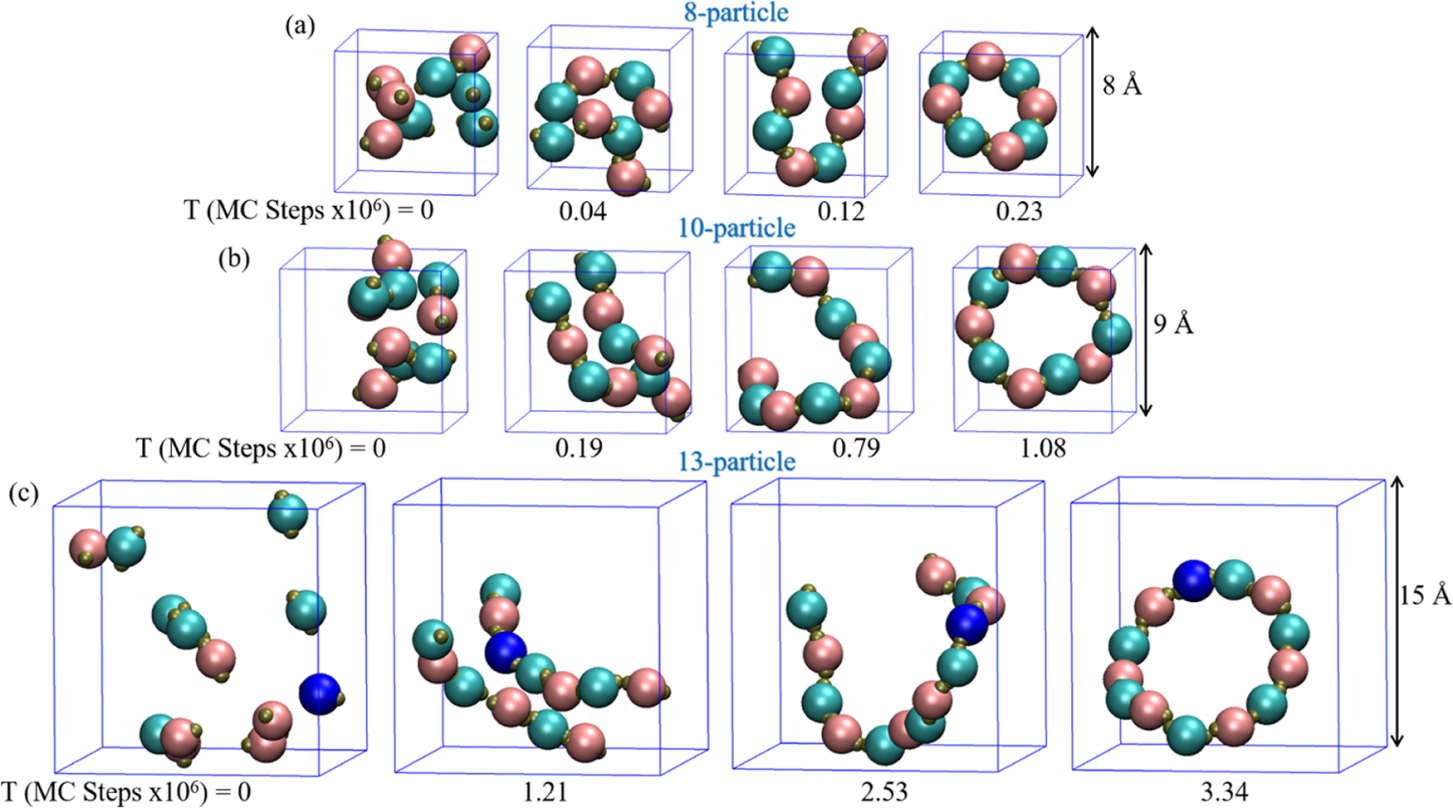
Equilibrium Monte Carlo simulation snapshots of (a) 8, (b) 10, and (c) 13 particles of type α (pink), β (cyan), and γ (blue), shown at various MC steps, starting from random initial conditions and ending at the assembly of the ring target structure. Sizes of the simulation boxes are shown.

In our study, we begin by conducting equilibrium Monte Carlo simulations for three different types of systems. The first two systems consist of 8 and 10 patchy particles with two internal states denoted by α and β. The third system consists of 13 patchy particles with three internal states denoted by α, β, and γ. Each realization is initiated at random configuration, aiming to self-assemble into the desired polygonal ring structure respective to its particle count. The assembly process is documented in snapshots (Figure 4), from the initial disorder to the final ordered state.

For the 8-particle system (Figure 4a), snapshots at initial, intermediate (0.04 × 10^6^ and 0.12 × 10^6^ MC steps), and final stages (0.23 × 10^6^ MC steps) illustrate a straightforward path to the target structure, indicating a simple energy landscape conducive to rapid assembly. The 10-particle system (Figure 4b) showcases a more gradual formation process. Initial and subsequent snapshots taken at 0.19 × 10^6^, 0.79 × 10^6^, and 1.08 × 10^6^ MC steps reveal an extended assembly time influenced by the larger particle count, underscoring the scaling challenges in self-assembly processes. The assembly of a 13-particle system (Figure 4c) is the most intricate, achieving the target state at 3.34 × 10^6^ MC steps after navigating through various stages. These snapshots collectively display the inherent complexity of each system and exemplify the typical pathway to the target configuration.

These configurations, captured at different MC steps and conducted under an interaction energy of 10 kJ/mol, highlight the kinetic pathways and the balance between entropic and energetic factors influencing assembly. As the number of particles increases, so does the complexity of the energy landscape, leading to longer assembly times. This emphasizes the potential for more intermediate metastable states in larger systems and the critical role of interaction energy in assembly efficiency. Next, we set to explore how the time to first assembly (*T*_fas_) and stability (*T*_stable_) depend on the patchy interaction potential (ϵ_patch_) across different system sizes.

#### 3.1.1 Time to First Assembly in Equilibrium MC Simulations

In an attempt to calculate the time to reach the first assembly from random configurations to understand the inherent trade-off of equilibrium simulations, we carry out MC simulation over a wide range of ϵ_patch_ values, ranging from 0 to 50 kJ/mol. A temperature of 65 K corresponds to 0.54 kJ/mol (*k*_B_*T*). Therefore, the range of ϵ_patch_, between 0 to 50 kJ/mol, translates to a range of 0 to 93 (ϵ/*k*_B_*T*). For each value of ϵ_patch_, the value of the time to first assembly, *T*_fas_, is quantized in MC steps.

**Figure 5:**
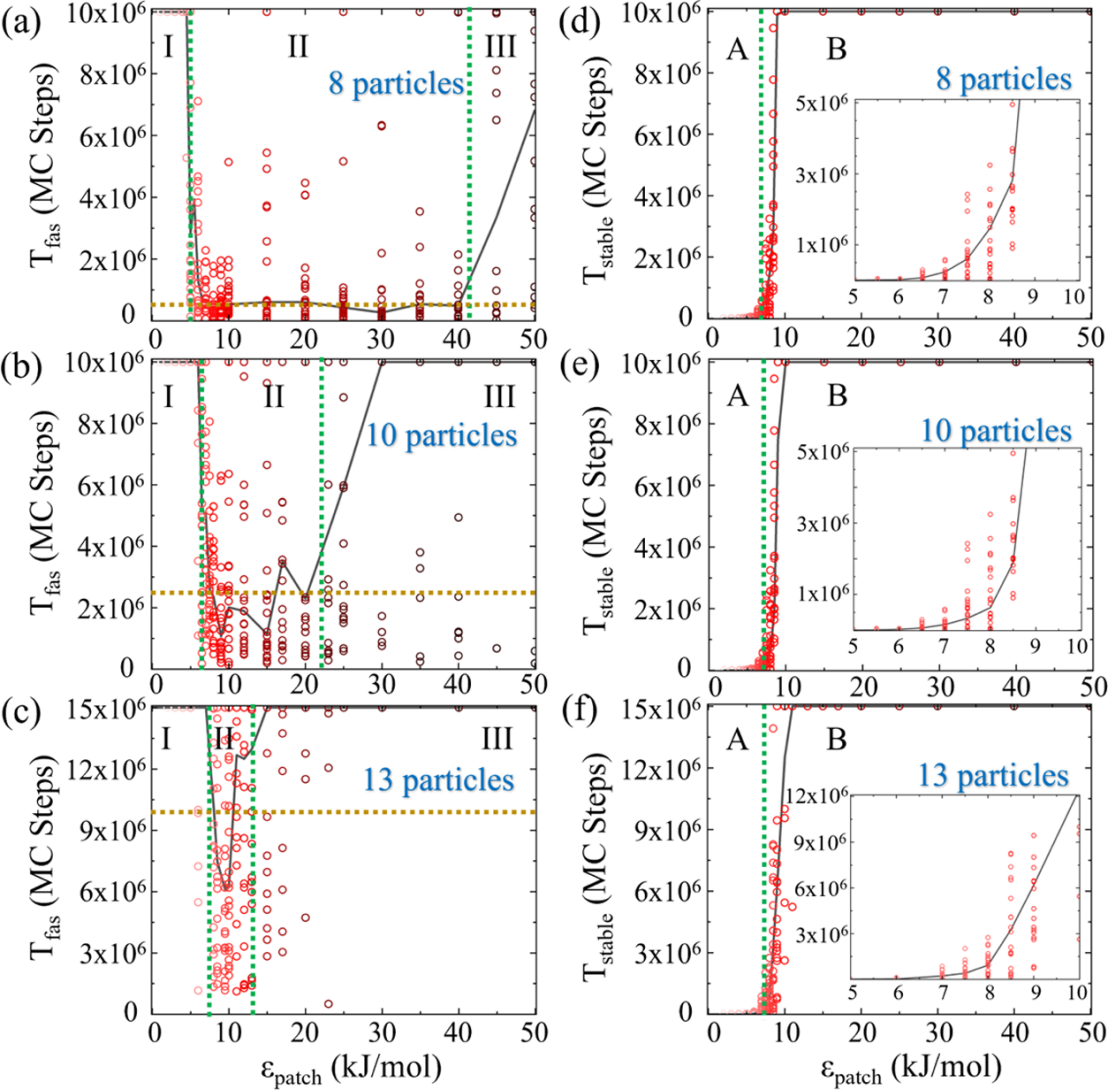
Equilibrium MC simulation results. Median *T*_fas_ values (black line) of 20 individual realizations (red circles), across different ϵ_patch_ values, for (a) 8 patchy particle system, (b) 10 patchy particle system, and (c) 13 patchy particle system. The ϵ_patch_ range is segmented into three regions (green dotted lines), with the average *T*_fas_ over the median values in the middle Region II depicted by a golden dotted line. Median *T*_stable_ values across different ϵ_patch_ values for (d) 8 patchy particle system, (e) 10 patchy particle system, and (f) 13 patchy particle system. The ϵ_patch_ range is categorized into two distinctive zones (green dotted lines). Insets: Zoom into the lower ϵ_patch_ range.

For the 8 particle (Figure 5a), 10 particle (Figure 5b), and 13 particle (Figure 5c) systems, the median value of *T*_fas_ remains relatively constant at the simulation length (10 × 10^6^ MC steps) at low interaction energies (ϵ_patch_), suggesting that particles do not coalesce into a stable assembly within the allotted simulation time, with ϵ_patch_ ranges of 0 to 5, 7, and 8 kJ/mol, respectively. These low ϵ_patch_ ranges which hinder assembly formation are referred to as Region I (see corresponding Movies S1, S2, and S3, for 8, 10, and 13 patchy particles in the SI).

As ϵ_patch_ increases and falls within and intermediate range, a notable decrease is observed in *T*_fas_. These ranges are 6–40 kJ/mol for the 8 particle system, 8–20 kJ/mol for the 10 particle system, and 8–12 kJ/mol for the 13-particle system. This decrease stabilizes to 0.7 × 10^6^, 2.1 × 10^6^, and 9.7 × 10^6^ MC steps for the 8, 10, and 13 patchy particle systems, respectively, as indicated by the golden dotted lines in Figure 5a–c. These values are obtained by averaging over the median values of *T*_fas_ in the intermediate Region II. The observed narrowing of this optimal interaction energy range with increasing system complexity from 8 to 13 patchy particles can be attributed to the intricate potential energy landscapes in larger assemblies. As the number of particles grows, the likelihood of encountering metastable states increases, necessitating a more precise tuning of interaction energies to navigate toward the desired assembly. This intermediate region (designated as Region II) is identified as the optimal energy regime for assembly at equilibrium, where the majority of simulations successfully reach the target structure, albeit with an increase in *T*_fas_.

At higher ϵ_patch_ values, *T*_fas_ begins to rise in all of the systems, suggesting that excessively strong interactions may be counterproductive, potentially trapping the system in kinetic traps. Significantly, for the 10 and 13 patchy particle systems, the median value of *T*_fas_ approaches the total number of MC steps, indicating more than half of the simulations fail to reach the target structure.

These trends across different interaction energy regimes highlight the delicate balance required for the self-assembly of patchy particles. Such insights are pivotal in advancing the fabrication of nanomaterials and the understanding of biological self-assembly processes where precise control over interaction energies is often beyond reach.

#### 3.1.2 Target Stability in Equilibrium MC Simulations

The target stability is represented by the time the system remains in the target structure, *T*_stable_. It is shown for systems comprising 8, 10, and 13 patchy particles in Figure 5d,e,f, respectively. At low ϵ_patch_ values (Region A), all systems display relatively low *T*_stable_, indicating insufficient stability of the target structures. As ϵ_patch_ increases to values larger than ∼6–7 kJ/mol (Region B), *T*_stable_ rises, indicating that the structures remain assembled throughout the entire simulation. For the 8-particle system, there is a sharp increase in *T*_stable_ beyond the critical threshold, while the 10 and 13-particle systems show a more gradual transition.

#### 3.1.3 Analysis of Assembly Time and Stability in Equilibrium Self-Assembly

The dynamics of assembly and stability across the 8, 10, and 13 patchy particle systems under equilibrium conditions are influenced by interaction energy, ϵ_patch_, as evident by the median values we have plotted. Plotting median values is necessary here due to the fact that in some regions of the parameter space, there were many realizations in which the target was not assembled (for *T*_fas_) or disassembled (for *T*_stable_), particularly in the case of low and high interaction energy values, which would bias the mean values. Therefore, showing the median ensures that the derived insights accurately reflect the typical system behavior. Low interaction energies prove insufficient for both assembly and stability across all systems, as indicated by the high values of *T*_fas_ (Region I) and low values of *T*_stable_ (Region A). At intermediate interaction energies, a clear decrease in *T*_fas_ (Region II) highlights the optimal range for self-assembly under equilibrium conditions. This range becomes narrower as system complexity increases from 8 to 10 and 13 patchy particles. In terms of target stability, beyond a ϵ_patch_ threshold, the assembled structure remains at the target state (Region B). Both *T*_fas_ and *T*_stable_ suggest a critical ϵ_patch_ range that balances the interaction energy necessary for assembly and subsequent stability, with larger systems requiring finer energy tuning to achieve and maintain assembled structures. At high interaction energies, an increase in *T*_fas_ (Region III) suggests that overly strong interactions may lead to kinetic traps and impede target structure formation. Concurrently, *T*_stable_ maintains its value equal to the simulation length for the higher values of ϵ_patch_.

In the realm of equilibrium self-assembly, the behaviors within Regions I and A, characterized by longer assembly times and low target stability, respectively, present a potential for external driving forces to play a critical role in accelerating the assembly while improving the stability of the targets. Such an intervention is particularly crucial in these regions, as it could overcome the limitations of equilibrium self-assembly, facilitating rapid assembly and longer stability. Furthermore, the range of interaction energy falling into the optimal region (Region II in Figure 5) for any system depends on the system complexity, including its size, the nature of interactions, the parameters of patches, and the number density.

#### 3.1.4 Time to First Assembly in Nonequilibrium MC Simulations

Nonequilibrium intervention in the form of an external drive has the potential to positively influence both the assembly speed and the target stability in Regions I and A, respectively. Here, we implement a driving force that favors the bond formation of nearest neighbor particles according to the target structure and disfavors bond breaking in this case.

**Figure 6:**
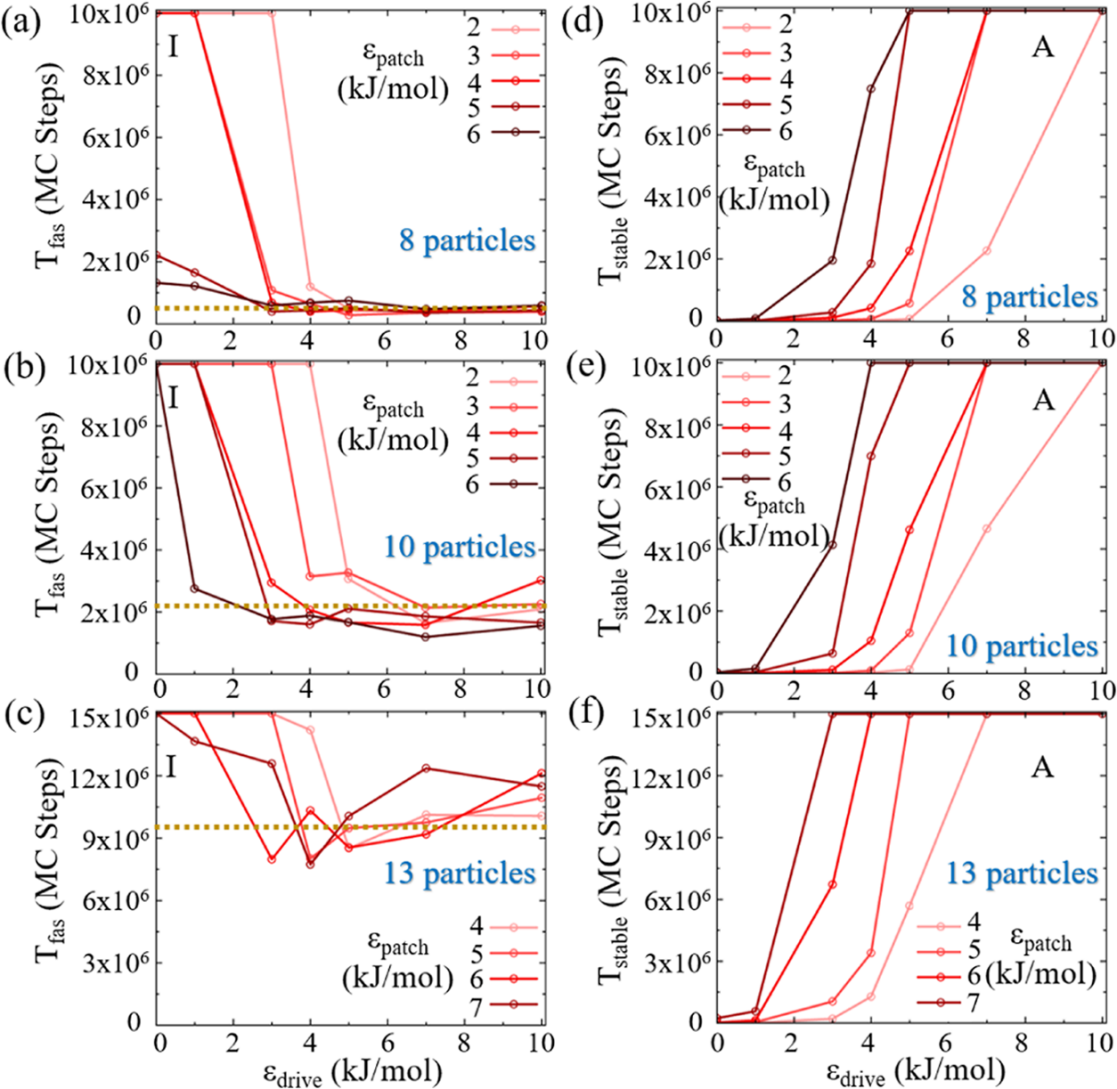
Nonequilibrium MC simulation results. Median *T*_fas_ values of 20 individual realizations for different patchy particle interaction energies, ϵ_patch_, as a function of the drive value, ϵ_drive_, for (a) 8 particle system, (b) 10 particle system, and (c) 13 particle system. The golden dotted line denotes the average value of *T*_fas_ in the intermediate Region II obtained from the corresponding results presented in Figure 5a–c. Median *T*_stable_ of 20 individual realizations for different patchy particle interaction energies, ϵ_patch_, as a function of the drive value, ϵ_drive_, for (d) 8 particle system, (e) 10 particle system, and (f) 13 particle system.

In the 8-particle system (Figure 6a) for ϵ_patch_ of 2 kJ/mol, *T*_fas_ remains at the maximum simulation length until ϵ_drive_ exceeds 3 kJ/mol, indicating that the system does not achieve the target structure without an adequate driving force. When ϵ_drive_ reaches 4 kJ/mol, *T*_fas_ is reduced significantly, suggesting that a specific threshold of an external drive is required to facilitate assembly. For ϵ_patch_ values of 3 and 4 kJ/mol, *T*_fas_ decreases to the equilibrium value of Region II (0.7 × 10^6^ MC steps) once ϵ_drive_ exceeds 3 kJ/mol. A higher value of ϵ_patch_ (5–6 kJ/mol) leads to a relatively small *T*_fas_ even without an external drive, highlighting the importance of intrinsic interaction energy in assembly efficiency. In the later cases, a minimal ϵ_drive_ of 1 kJ/mol is adequate to reduce *T*_fas_ to its lowest observed plateau. To achieve similar reductions in *T*_fas_ for the 10-particle system, converging to the average *T*_fas_ value of the corresponding Region II (Figure 5b), a higher ϵ_drive_ of 5 kJ/mol is required for ϵ_patch_ value of 2 kJ/mol. As ϵ_patch_ increases from 3 to 6 kJ/mol, lower ϵ_drive_ is required.

For the 13-particle system (Figure 6c), *T*_fas_ remains at the simulation length value (15 × 10^6^) without the intervention of external drive for all ϵ_patch_ values. An external drive of 4 kJ/mol is needed at ϵ_patch_ = 4 kJ/mol to reach the target, with higher ϵ_drive_ reducing *T*_fas_ to the equilibrium average value of Region II (Figure 5c). As ϵ_patch_ increases from 5 to 7 kJ/mol, lower ϵ_drive_ values are needed.

Comparing the various system sizes, for ϵ_patch_ value fixed at 6 kJ/mol, a drive value ϵ_drive_ of 3 kJ/mol is required to reduce *T*_fas_ to its equilibrium value in the 13 particle system, where only 1 kJ/mol is required to achieve the same result in a 10 particle system, and the 8 particle system archives the equilibrium value without any external drive.

These simulations show that external drives can effectively reduce the assembly times up to a point where an increase in the driving force does not result in additional acceleration. This suggests that while external driving forces can enhance assembly speed, there exists a limit to their efficacy beyond which the system behaves similarly to the equilibrium systems observed in the corresponding Region II. For a visualization of the nonequilibrium self-assembly process, see Movies S4, S5, and S6, for 8, 10, and 13 patchy particles, respectively, in the SI.

#### 3.1.5 Target Stability in Nonequilibrium MC Simulations

The external drive also increases the target stability, manifested in higher *T*_stable_ for increasing drive values for several ϵ_patch_ values within the corresponding Region A for all systems tested, of 8 (Figure 6d), 10 (Figure 6e), and 13 (Figure 6f) patchy particles, aligning with previous findings.^96,98,99^

In the 8-particle system (Figure 6d), at ϵ_patch_ = 2 kJ/mol, *T*_stable_ gradually increases with ϵ_drive_, aligning with the simulation length (10 × 10^6^ MC steps) beyond 5 kJ/mol. As ϵ_patch_ increases from 3 to 6 kJ/mol, lower ϵ_drive_ values suffice to enhance *T*_stable_, indicating higher internal interaction energies improve stability. For the 10-particle system (Figure 6e), *T*_stable_ at ϵ_patch_ = 2 kJ/mol increases only beyond ϵ_drive_ = 6 kJ/mol, with higher ϵ_patch_ reducing the needed ϵ_drive_. In the 13-particle system (Figure 6f), at ϵ_patch_ = 4 kJ/mol, ϵ_drive_ = 3 kJ/mol significantly increases stability. For higher ϵ_patch_ values, modest increases in ϵ_drive_ ensure maximal stability.

#### 3.1.6 Analysis of Nonequilibrium Self-Assembly Mechanism

We now turn to explore the assembly dynamics of patchy particles under external forces by focusing on an order parameter (*R*) and the total entropy production (*S*)^122^ as a function of MC steps. *R* is defined as the ratio of the number of formed bonds at any given MC step to the total number of bonds in the target structure, therefore quantitatively characterizes the path to assembly. The detailed description of the computation of *S* in our MC simulation is provided in Section S1 of the Supporting Information (SI).

**Figure 7:**
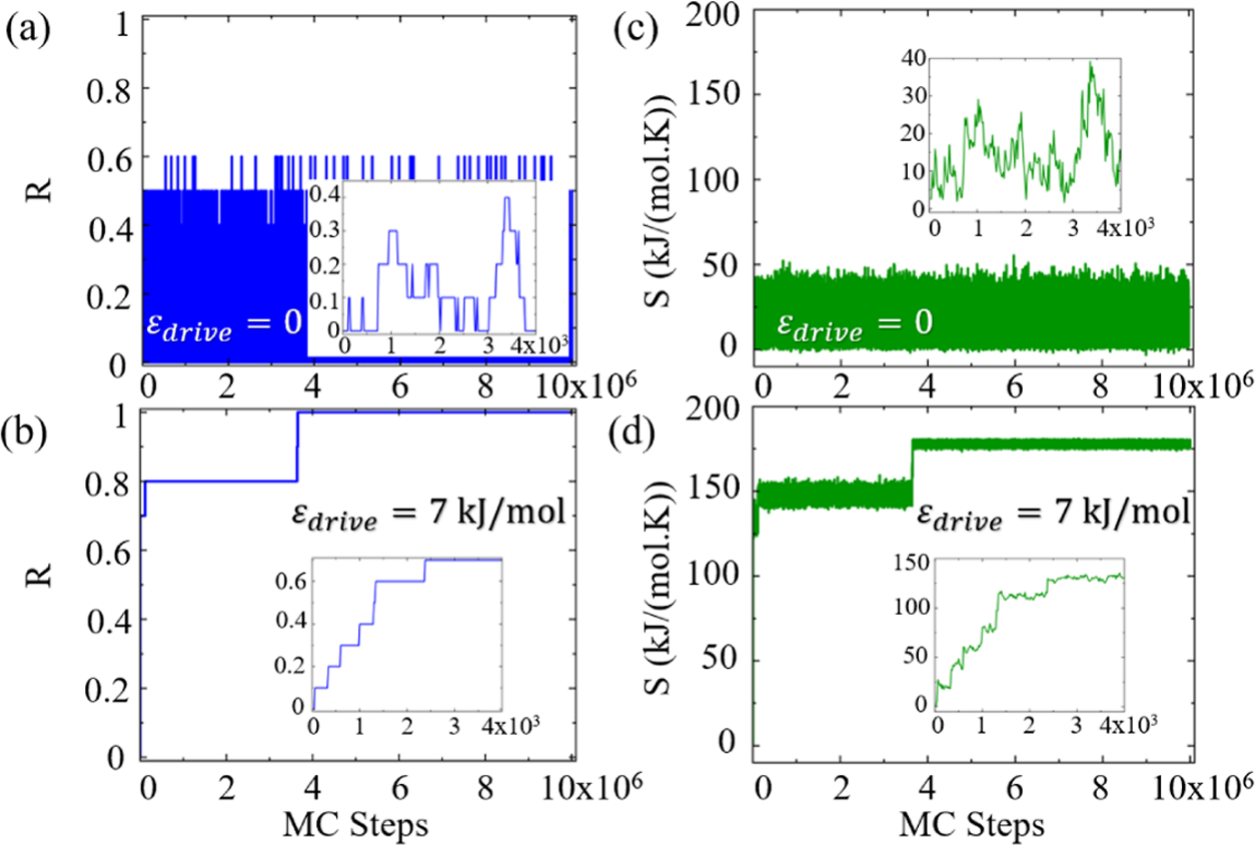
Order parameter (*R*) as a function of MC steps (a) in equilibrium and (b) under nonequilibrium conditions with an external drive ϵ_drive_ = 7 kJ/mol. The total entropy production (*S*) as a function of MC steps (c) in equilibrium and (d) under nonequilibrium conditions with an external drive ϵ_drive_ = 7 kJ/mol Results are presented for a single MC realization of 10 particle system with ϵ_patch_ = 3 kJ/mol. The inset plots show Zoom in into the early simulation steps.

The evolution of *R* for a 10 patchy particle system with patch interaction value of ϵ_patch_ = 3 kJ/mol, under equilibrium, ϵ_drive_ = 0, and nonequilibrium conditions, ϵ_drive_ = 7 kJ/mol, is depicted in Figure 7a and b, respectively. In the absence of an external drive, *R* displays stochastic behavior, oscillating between 0 and 0.6, manifesting the inability of the system to assemble the target. With the external drive, a stark transition in the behavior of the order parameter is observed, with *R* ascending from a disordered state directly toward the fully assembled target structure, corresponding to *R* = 1 at 3.7 × 10^6^ MC steps. The quantized changes in *R* represent the discrete steps of the subsequent formation of 10 bonds, ultimately yielding the 10 particle ring structure.

Similarly, the total entropy production, *S*, exhibits fluctuations at equilibrium (Figure 7c), indicating vanishing entropy production rate, as expected.^122^ Under nonequilibrium conditions, the value of *S* rapidly increases (Figure 7d), reflecting the dissipation of energy accompanying the utilization of the external drive.

The contrast in the behavior of *R* and *S* with and without an external driving force emphasizes the significant role of external driving forces in directing the thermodynamic path of patchy particle systems. The external drive enhances the order within the system at the cost of the production of entropy.^123^ Similar observations for trajectories of 8 and 13 patchy particles in equilibrium and nonequilibrium MC simulations can be found in Figures S1 and S2 in Section S2 of the SI.

**Figure 8:**
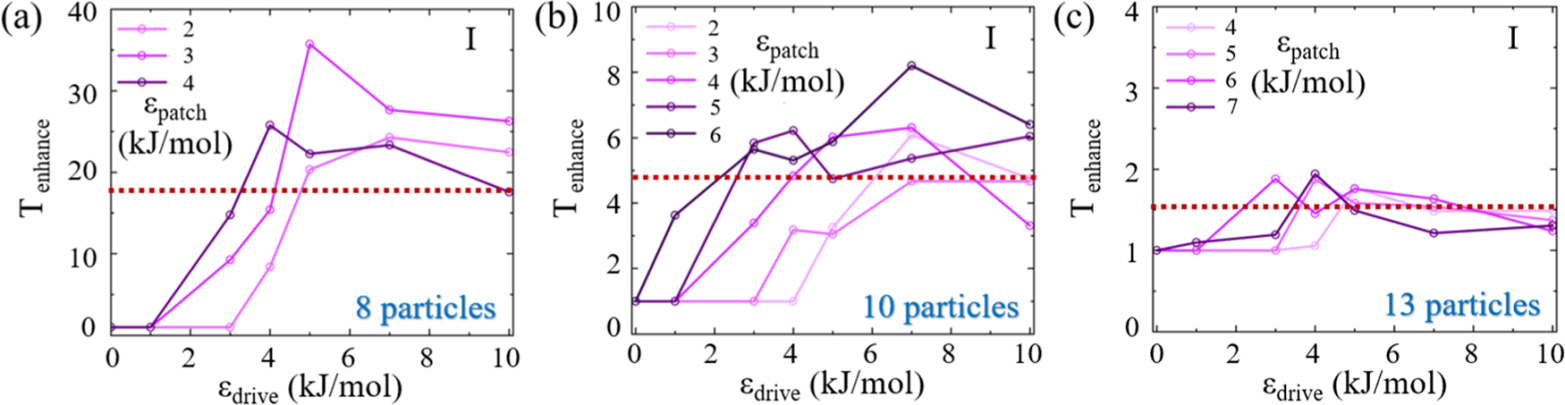
*T*_enhance_ as a function of ϵ_drive_ for various ϵ_patch_ values from Region I for systems of (a) 8 particle, (b) 10 particle, and (c) 13 particle systems. The brown dotted line represents the ratio of *T*_fas_ between Regions I and II, obtained from the corresponding results in Figure 5a–c.

The effect of the external drive is quantified by *T*_enhance_, defined as the ratio of the median time to the first assembly with the external drive (ϵ_drive_) to the median time without the drive, across a set of 20 distinct simulations for a given value of ϵ_patch_. This metric provides a measure of the efficiency improvement in assembly time attributable to the external drive. For example, this metric for 8 particles is obtained from the values plotted in Figure 6a for a given system. For the 8 particle system (Figure 8a), *T*_enhance_ shows a significant increase with increasing ϵ_drive_, showing approximately 35 fold improvement in the assembly time compared to the equilibrium assembly for ϵ_patch_ = 3 kJ/mol, for example. In the 10 particle system (Figure 8b), an increase in *T*_enhance_ with rising ϵ_drive_ is observed, with more modest improvement in assembly time. The enhancement reaches a plateau at about 6 to 8 fold improvement, occurring at an ϵ_drive_ near 6 kJ/mol. The variation of *T*_enhance_ of 13 particle system is displayed in Figure 8c, achieving a moderate improvement of up to 2-fold in the assembly time compared to the equilibrium scenario. Compared to the increase of *T*_enhance_ for 8 and 10 particle systems with the increase in drive, the increment for the 13-particle system does not show similar improvement with increasing the drive value. This reflects the more complex assembly pathway involving 13 patchy particles and multiple interaction types: α – β, α – γ, and β – γ, compared to only α – β interaction between the other two smaller systems. The enhancement factors *T*_enhance_ are presented along the ratio of *T*_fas_ from Region I to *T*_fas_ in Region II in equilibrium simulations (Figure 8, dotted lines), further indicating that the drive helps the system achieve its target within the time frame that matched the one of the optimal region (Region II).

In order to test the effects of our chosen patch size and particle density parameters,^76,124^ we explore the time to first assembly and target stability for various values of the length of the patch, δ/2 (Section S3 in the SI) and different lengths of the simulation cell (Section S4 in the SI). Importantly, the trend of *T*_fas_ and *T*_stable_ under both equilibrium and nonequilibrium conditions remains similar, and our conclusions hold for the different cases.

### 3.2 Molecular Dynamics Simulation Results

Transitioning into the MD simulations, we expand upon the findings from the MC simulations to further explore the dynamic aspects of dissipative self-assembly of the patchy particle system under both equilibrium and nonequilibrium conditions. MD simulations, with their capacity to capture real-time dynamics, are instrumental in providing a more thorough understanding of the mechanisms at play. This approach complements the MC results by offering insights into the temporal evolution of particle assemblies, thereby enriching our analysis with a perspective that closely mirrors physical realities.

Moreover, the implementation of nonequilibrium MD simulations is a deliberate effort to bridge the computational findings with potential experimental validations. By examining the system’s response to external drives and the resultant dynamic behaviors, this segment aims to not only complement the MC-derived thermodynamic landscapes but also to lay a foundational basis for future experimental investigations.

#### 3.2.1 Equilibrium MD Simulations

We first set to explore the time to reach the target structure starting from random initial conditions within the simulation box for systems of 8 and 10 patchy particles with a single internal state, α. The time to first assembly, *T*_fas_, is calculated over a broad range of interaction energies between patches, ϵ_patch_, ranging from 0 to 100 kJ/mol. Similar to the approach taken in our equilibrium MC simulations (refer to Section 3.1.1), the behavior of *T*_fas_ as a function of ϵ_patch_ can be categorized into three distinct regions. At low ϵ_patch_ values, the system is unable to achieve the target structure within the allotted simulation time of 12 000 ps due to the weak interaction (Region I), whereas for larger ϵ_patch_ values, *T*_fas_ decreases, manifesting an optimal range for assembly (Region II). Beyond a particular ϵ_patch_ value, *T*_fas_ begins to rise, signifying the onset of kinetic trapping that inhibits the particle reorientation toward the target structure (Region III).

**Figure 9:**
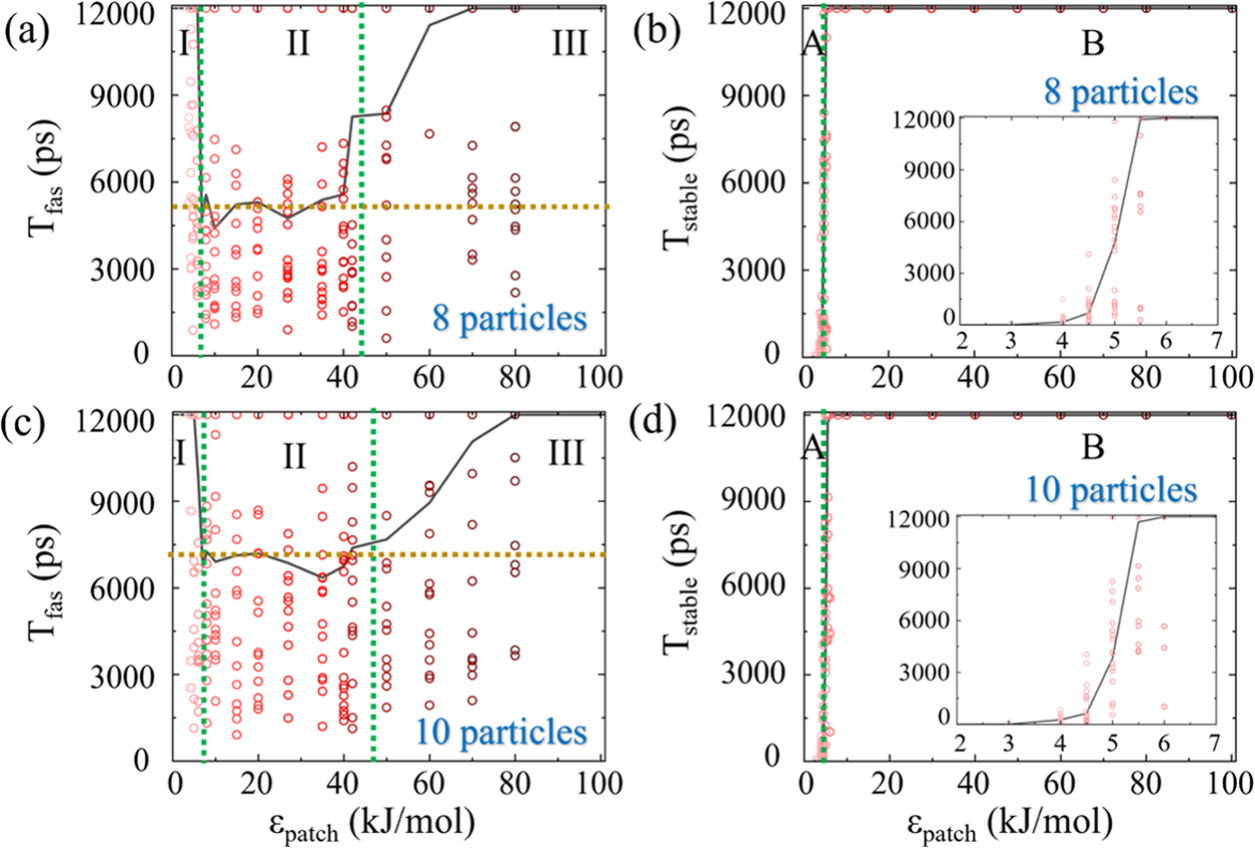
Equilibrium MD simulation results. Median *T*_fas_ values (black line) of 20 individual realizations (red circles), across different ϵ_patch_ values, for (a) 8 patchy particle system and (b) 10 patchy particle system. The ϵ_patch_ range is segmented into three regions (green dotted lines), with the average *T*_fas_ over the median values in the middle Region II depicted by a golden dotted line. Median *T*_stable_ values across different ϵ_patch_ values for (c) 8 patchy particle system, and (d) 10 patchy particle system. The ϵ_patch_ range is categorized into two distinctive zones (green dotted lines). Insets: Zoom into the lower ϵ_patch_ range.

For the 8 particle system (Figure 9a), no assembly occurs for ϵ_patch_ values between 0 to 6 kJ/mol (Region I). Beyond 6 kJ/mol, *T*_fas_ decreases and stabilizes at 5115 ps (Region II), until approximately 40 kJ/mol (Region III), where *T*_fas_ increases up to the simulation duration (Movie S7 in the SI). Similar results with 8 patchy particles with hard-sphere interaction were observed in our concurrent study,^123^ where *T*_fas_ transitioned from Region II to III above 40 kJ/mol value of ϵ_patch_.

In addition to the time to reach the assembled state, we set to study the stability of the resulting target structure by the time the system remains at the target state, *T*_stable_, in a set of simulations initiated at the target across the same ϵ_patch_ range. As for the MC simulations (Section 3.1.2), the behaviors of *T*_stable_ clearly segment the ϵ_patch_ values into the regions, where for low ϵ_patch_ values the target quickly disassembles (Region A), whereas for higher values, the target remains stable (Region B).

In the case of the 8 particle system (Figure 9b), for ϵ_patch_ values up to 4 kJ/mol, *T*_stable_ is minimal (a few ps), indicating low system stability. However, as ϵ_patch_ increases, the median *T*_stable_ rises and reaches the full duration of the simulation (12 000 ps) by ϵ_patch_ = 6 kJ/mol. These observations lead to the classification of Region A to include ϵ_patch_ values between 0–6 kJ/mol, and Region B between ϵ_patch_ values of 7–100 kJ/mol.

The same methodology is applied to a system of 10 patchy particles with a single internal state, α. The trend of *T*_fas_ as a function of ϵ_patch_ mirrors the behavior of the 8 patchy particle system (Figure 9c), and the ϵ_patch_ range was similarly segmented into Region I (0–6 kJ/mol), Region II (7–50 kJ/mol), and Region III (50–100 kJ/mol). The average of median *T*_fas_ in Region II is 7024 ps, shown as a golden dotted line, which is slightly higher than that of the 8 patchy particle system owing to its lower complexity. As for the stability of the resulting target quantified by *T*_stable_ (Figure 9d), we observe low target retention of only a few ps for low ϵ_patch_ values (Region A). When ϵ_patch_ exceeds 4 kJ/mol, the median *T*_stable_ rises and reaches the full simulation duration (12 000 ps) at 6 kJ/mol (Region B), (See Movie S8 in the SI).

As argued in Section 3.1.3, Regions I and A are characterized by prolonged assembly times and reduced structural stability, respectively, thereby presenting an opportunity for an external bias to overcome these equilibrium limitations and to enable quicker self-assembly and improved target stability.

#### 3.2.2 Nonequilibrium MD Simulation Results

In nonequilibrium MD simulations, we explore the influence of an external drive in the form of an external square wave potential with varying amplitude, *U*_square_(*t*), added to the patchy interaction potential, *U*_patch_(*r*), on the assembly dynamics for systems of 8 and 10 patchy particles. The amplitudes of *U*_square_(*t*) ranges from 0 to 60 kJ/mol, for patchy interaction ϵ_patch_ values of 4.5, 5, and 6 kJ/mol from Regions I and A. For each scenario, we determine the time to the first assembly, *T*_fas_, and the target stability, *T*_stable_.

**Figure 10:**
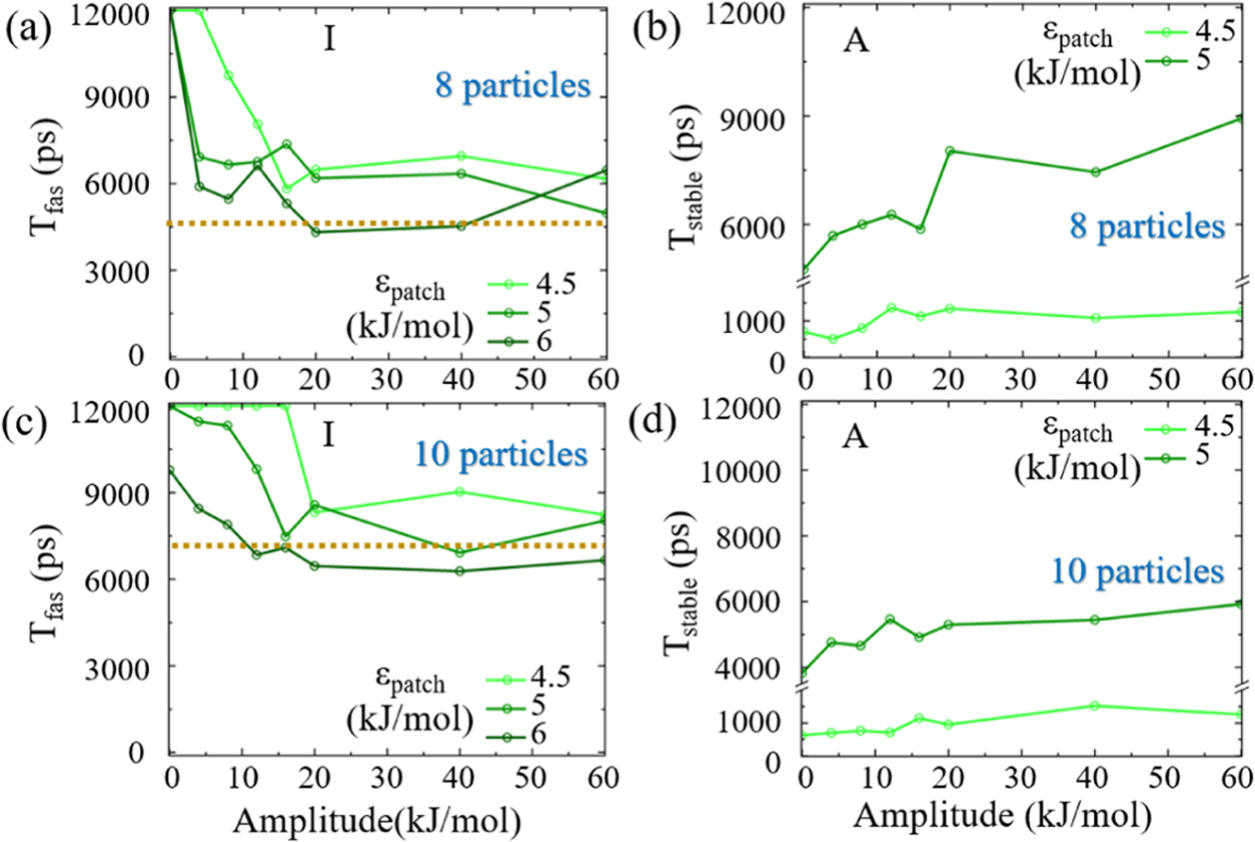
Nonequilibrium MD simulation results. Median *T*_fas_ values of 20 individual realizations for different patchy particle interaction energies, ϵ_patch_, as a function of the amplitude of the square wave potential for (a) 8 particle system, and (c) 10 particle system. The golden dotted line denotes the average value of *T*_fas_ in the intermediate Region II obtained from the corresponding results presented in Figure 9a and b. Median *T*_stable_ of 20 individual realizations for different patchy particle interaction energies, ϵ_patch_, as a function of the amplitude of the square wave potential for (b) 8 particle system, and (d) 10 particle system.

For 8 particle system with ϵ_patch_ = 4.5 kJ/mol, *T*_fas_ significantly reduces for external drive amplitudes above 4 kJ/mol, where for amplitude value of 20 kJ/mol and beyond, *T*_fas_ drops to around 6000 ps (Figure 10a). For ϵ_patch_ values of 5 and 6 kJ/mol, *T*_fas_ immediately shows a significant decrease to around 6000 ps (Movie S9 in the SI). This value is similar to the average *T*_fas_ of approximately 5100 ps obtained in Region II of 8 patchy particle equilibrium MD simulations (Figure 9a, golden dotted line).

In terms of stability of 8 particle system, for ϵ_patch_ of 4.5 kJ/mol, *T*_stable_ is 700 ps at zero amplitude (Figure 10b), and increases to 1200 ps as the amplitude of the external drive increases to 12 kJ/mol and further. At 5 kJ/mol, *T*_stable_ rises from 4000 to 9000 ps with an increasing amplitude of up to 60 kJ/mol. These results indicate that the introduction of an external square wave potential can substantially enhance stability for the 8 patchy particle system.

To further complement our study, we conduct MD simulations for a 10 patchy particle system, considering the influence of an external square wave potential. At ϵ_patch_ = 4.5 kJ/mol, *T*_fas_ remains largely unchanged until the potential amplitude exceeds 20 kJ/mol, then fluctuates around 7500 ps (Figure 10c, Movie S10 in the SI). At ϵ_patch_ = 5 kJ/mol, *T*_fas_ decreases from 12 000 to 9000 ps at 16 kJ/mol amplitude, and further to 7000 ps at 60 kJ/mol. For ϵ_patch_ = 6 kJ/mol, *T*_fas_ decreases from 10 000 to 6000 ps as the amplitude increases to 12 kJ/mol, suggesting a greater sensitivity of the assembly time to the applied potential in comparison to the lower ϵ_patch_ values. These findings align with the average *T*_fas_ values observed in Region II of the equilibrium MD simulations for the 10 patchy particle system (Figure 9c, golden dotted line).

The stability of the 10 patchy particle system under nonequilibrium conditions is analyzed by varying the amplitude of the external drive while initiating the system at target under ϵ_patch_ values of 4.5 and 5 kJ/mol (Figure 10d). The value of *T*_stable_ increases from 520 to 1100 ps as the amplitude is increased from 0 to 16 kJ/mol, where further increment in amplitude does not result in an additional increase in *T*_stable_. For ϵ_patch_ value of 5 kJ/mol, the stability time *T*_stable_ rises from 4000 to 6000 ps with a modest amplitude increment to 10 kJ/mol. Similarly, subsequent increases in the amplitude do not enhance *T*_stable_ significantly.

Comparing the 8 and 10 patchy particle systems in nonequilibrium MD conditions, we notice distinct responses to the external drive. Both systems experience reduced assembly times (*T*_fas_) as the amplitude of the square wave potential increases. The 10 patchy particle system, however, shows a higher sensitivity to the amplitude changes, necessitating finer tuning in driving forces for comparable reductions in *T*_fas_. This size-dependent response highlights how system complexity affects the dynamics of self-assembly, similar to our observation in the nonequilibrium MC simulations. Further, stability analysis (*T*_stable_) reveals that while the stability improves for both systems with higher drive amplitudes, the enhancement is less pronounced in the 10 patchy particle system. In contrast to the nonequilibrium MC simulations, where the stability (*T*_stable_) is maintained throughout the entire simulation, the nonequilibrium MD simulations reveal further opportunities for improvement. This suggests that optimizing the effectiveness of the square wave potential as an external driving force can potentially further increase target stability.

The acceleration of the assembly and the improved target stability we find in our MD simulations are also manifested in the analysis of the order parameter, *R*, defined in a similar way as for the MC simulations, as the ratio between the number of formed bonds to the total number of bonds in the target structure. We calculate the value of *R* averaged over the entire simulation duration and observe a moderate increase when the time-dependent square wave potential is introduced to the system, for systems of 8 and 10 particles (Section S5 in the SI).

In our investigation of assembly kinetics and stability under nonequilibrium conditions using MD simulations, we meticulously analyze the behavior of bond formation and dissociation events across both high and low energy phases induced by the square wave potential, *U*_square_(*t*). From this analysis, we observe that the bond durability in our patchy particle system is enhanced with the increase of square wave amplitudes, which in turn boosts the stability of the target structure and aids in its assembly (Section S6 in the SI).

#### 3.2.3 Potential Energy and Force Comparison of 2 Particle System

In order to better understand the dynamics of bond formation, we conduct a set of MD simulations of a system comprising only 2 particles randomly initiated up to the first bond formation event in equilibrium and nonequilibrium conditions. During the simulation realizations, we track the total potential energy of the system and the force between the particles. For this study, the simulation cell dimensions are set to 2 × 6 × 6 Å^3^, with patch parameters identical to those in a 10 particle system. Apart from the simulation cell size, all simulation parameters are consistent with those used in simulations of the 8 and 10 patchy particle systems. Importantly, the outcomes do not strictly depend neither on the dimensions of the simulation cell nor on the patch parameters (δ/2 and θ^max^).

**Figure 11:**
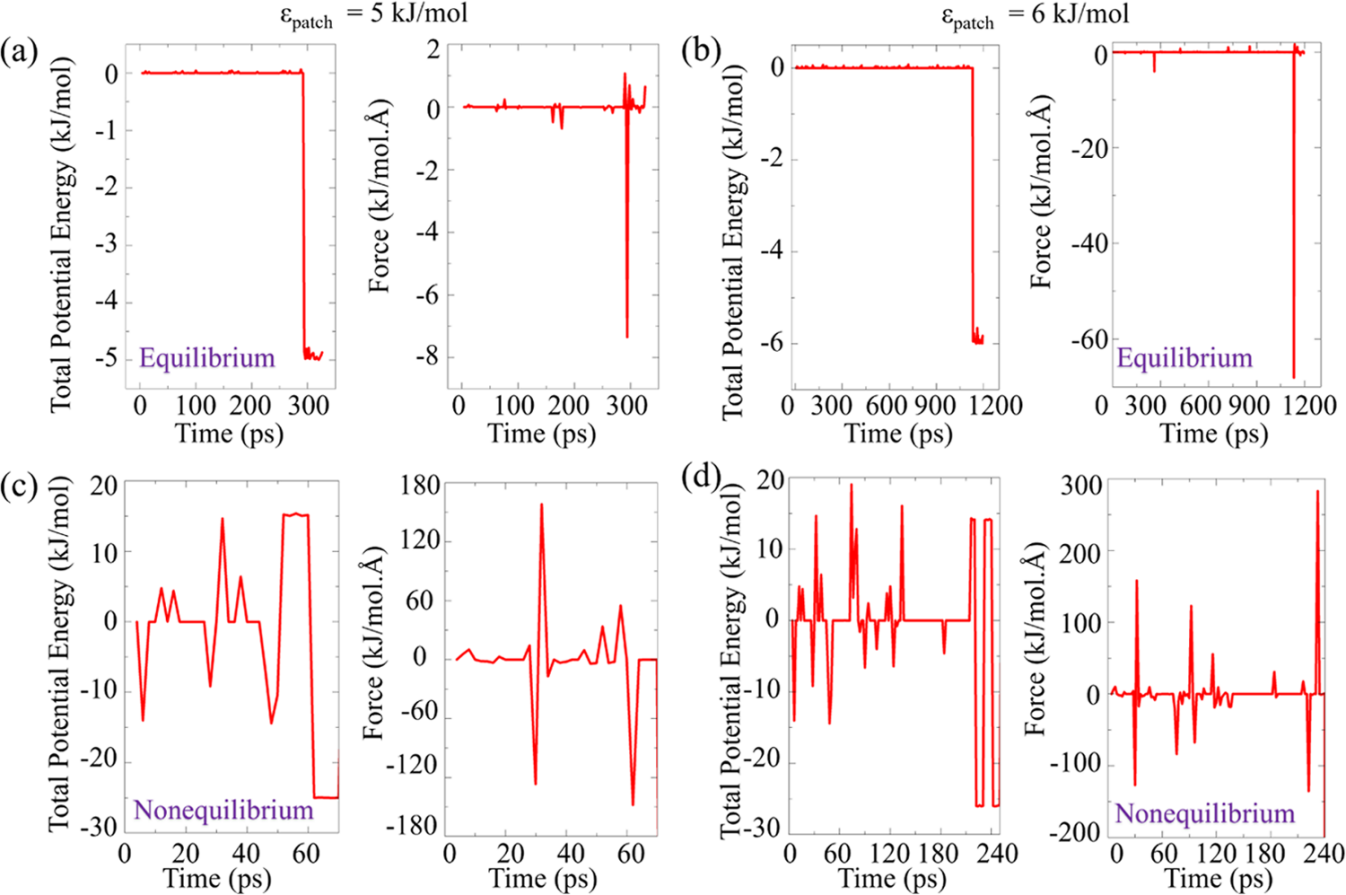
MD simulation of a 2 particle system up to the first bond formation event. Total potential energy and force as a function of time for equilibrium conditions with ϵ_patch_ value of (a) 5 kJ/mol and (b) 6 kJ/mol, and nonequilibrium conditions with square wave potential amplitude of 20 kJ/mol for ϵ_patch_ value of (c) 5 kJ/mol and (d) 6 kJ/mol, shown for individual realizations.

Under equilibrium conditions for patchy interaction energies ϵ_patch_ of 5 kJ/mol (Figure 11a) and 6 kJ/mol (Figure 11b), the potential energy initially fluctuates around 0, and the corresponding force between the particles, reflecting the gradient of the potential energy, also remains close to 0. On the other hand, the bond formation event, which occurs at 294 and 1132 ps for ϵ_patch_ of 5 and 6 kJ/mol, respectively, is manifested in a noticeable decrease of the potential and a concurrent force spike.

In stark contrast, nonequilibrium conditions exhibit a completely different profile of the total potential energy and the force for both patchy interaction energies of 5 kJ/mol (Figure 11c) and 6 kJ/mol (Figure 11d). Here, the potential energy is characterized by significantly larger fluctuations owing to the varying high and low energy phases of square wave potential, resulting in larger forces acting between the two particles compared to the corresponding equilibrium values. The particles are, therefore, subject to frequent and substantial energy changes, driving them to reorient and adjust their positions as they seek stable configurations. The difference in the force magnitudes between equilibrium and nonequilibrium underscores the mechanism of the increased bond-formation events observed in Figure S7. These larger forces in the nonequilibrium state catalyze the movement of particles toward each other, expediting the bonding process.

### 3.3 Effect of External Drive on Self-Assembly of Large Systems

In order to demonstrate the efficacy of our proposed design principle of applying an external nonequilibrium drive for a large-scale system, we ran both equilibrium and nonequilibrium simulations of 100 patchy particles (Section S7 in the SI). The particles were modeled with PHS interaction between patches and PBC of the simulation cell to examine how interaction energies ϵ_patch_ and ϵ_drive_ influence the structure formation. The patches in these simulations were positioned with an interior angle of 135° for 8-ring formations and 90° for 4-ring formations to explore the ring formation in the resultant assembly structure subjected to different patch orientations, using other parameters similar to those in smaller-scale systems of 8 patchy particles.

The equilibrium (Figure S8a) and nonequilibrium (Figure S8b) results for the larger system of 100 particles are consistent with the observations of our smaller systems of 8, 10, and 13 patchy particles, showing faster bond formation in the presence of the drive compared to the corresponding equilibrium results with similar interaction strength between the patches. Similar observations have been showcased in Figure S9a and b for large systems with 4-ring specifications. The overall results are consistent with the small-scale system, demonstrating that using an external drive can facilitate a faster assembly process in the region of weak ϵ_patch_. Thus, our approach of nonequilibrium drive is independent of the overall system size, effectively negating any finite-size effects and emphasizing the robustness of our assembly protocol.

It is important to note that the formation of rings strictly depends upon the system parameters, particularly on the patch orientation and the number density of the system. While our specific system with a patch orientation corresponding to an 8-ring structure produces open rings or chains (Movie S11), the systems with a patch orientation of a 4-ring, as used in our work, produce rings (Figure S9c and Movie S12). Moreover, adding an external drive to the system in the latter case increases the number of rings formed for weak patchy interactions where no structure formation occurs without the drive (Figure S9d), similar to our previous observations.

We also conducted an equilibrium MD simulation of a 4-ring specific system comprising 100 particles, utilizing PHS interactions and PBC for the simulation cell (Figure S10). This simulation corroborated the structural formations observed in our MC simulation results, as shown in Figure S8a, thereby validating the consistency and reliability of our findings across different simulation methodologies.

Since our external drive depends only on the interaction between patches that are nearest neighbors in the predefined target structure, it can only accelerate the structure formation observed in equilibrium simulations, as the nature of the formed structure strictly depends on internal system parameters. Additionally, while increasing the specificity of patchy interactions can impact the optimal interactions for assembly rate and stability time scales, it has no effect on the mechanism of the external drive employed in our design. Therefore, a careful balance of specificity and robustness is necessary for optimal self-assembly processes.

## 4 Conclusions

In our study, we embarked on an exploration to discern the limitations posed by equilibrium conditions in self-assembly processes, and how external drives could alleviate these challenges. Motivated by both biological self-assembly processes (particularly of actin filament and microtubules) and experimental observations, we introduce an external stimulus to a model system of self-assembling patchy particles, to study the resulting dissipative self-assembly. To generalize the findings from this study in the future to specific biological systems, patchy particles have been employed here as it has already been shown to closely mimic the dynamic behavior of actin filament and microtubule structure.^125,126^ Through a synergistic approach combining both MC simulations and MD simulations, under equilibrium and nonequilibrium conditions, our objective was to elucidate the impact of the nonequilibrium external drive on the dynamics of the system. This involved a detailed examination of trajectory realization and an analysis of the fundamental mechanisms driving the observed behaviors.

Our findings reveal that the equilibrium MC simulations of patchy particles with 8, 10, and 13 particles exhibit low assembly rates and low structural stability for weak patchy particle interactions. The external drive greatly improved the time to assembly and increased the structural stability of the target for the weak interactions regime, reaching the optimal values obtained at equilibrium for a narrow range of the patch interaction energies. This extends the ability of the system to assemble the target for a wider range of conditions compared to the equilibrium scenario, owing to the nonequilibrium external drive.^96,98,99^ The order parameter (*R*) and total entropy production (*S*) along with the realizations demonstrated how the external drive guided the system from a disordered initial state to the order configuration of the target structure.

Our detailed investigation revealed that the complexity of the system influences the self-assembly process in both equilibrium and nonequilibrium conditions. As the complexity increased to a larger number of particles in the system, the effect of the drive was smaller in terms of the final assembly rates and structural stability. We hypothesize that as complexity increases, the multitude of potential interaction pathways and configurations may lead to kinetic trapping or interference, thereby diminishing the influence of the external stimuli. Consequently, this emphasizes a substantial opportunity for improving the design principles of incorporating external stimuli, ensuring they are dynamically responsive to the increasing complexity of the system. Furthermore, the impact of patch length and number density on the self-assembly dynamics of patchy particles had no significant effects on the assembly time and structural stability of the target.

To further extend our conclusions, we employed MD simulations of self-assembling patchy particles with a periodic square wave potential as an external drive, inspired by the experimental applications of varying external fields.^84,85^ This approach demonstrated similar trends in the acceleration of the assembly and the increase in target stability, mirroring our MC simulation results. While the effect of the drive brought the system to the optimal performance level at equilibrium, its importance was in the ability to allow for rapid assembly and increased stability in a wider parameter range, i.e., lower values of the patch interaction energy, not possible in equilibrium without the external driving. Furthermore, the dynamic properties such as translational and rotational diffusivity are influenced by the mass of the particles and patches, but the relative behavior between equilibrium and nonequilibrium conditions remains qualitatively consistent regardless of the mass ratio. Additionally, an analysis of particle forces and bond dynamics in the presence of the nonequilibrium drive indicated that large momentary forces were responsible for bond formation and stability. Future efforts will include a comprehensive sensitivity analysis of additional system parameters, including the number of particle states, angular dependencies, and varying boundary conditions, to fully understand their impact on self-assembly dynamics.

Our study contributes to the understanding of how external drives can be strategically employed to circumvent the equilibrium limitations in self-assembly processes. By employing the LJ potential with different internal states and reflective boundary conditions in MC simulations and the PHS potential with a single internal state and PBC in MD simulations for smaller systems (8, 10, and 13 patchy particles), we demonstrate the versatility and effectiveness of our design protocol across varying conditions. This approach highlights the robustness of our protocol in achieving faster and more stable assembly, regardless of the simulation method employed. Additionally, to ensure that the choice of boundary conditions does not significantly affect our results, we conducted simulations of 100 particles using PBC in both MC and MD simulations. The outcomes showed similar results, indicating that the qualitative behavior of assembly remains consistent across different boundary conditions. The use of a square wave as a model for external stimuli opens avenues for experimental implementation, offering insights into experimental observations and applications of patchy particles, which can be implemented across various systems, such as colloids and block copolymers.^22,65,71^

Looking forward, a deeper understanding of dissipative self-assembly mechanisms will push the development of novel synthetic nanostructures for numerous applications. Furthermore, the effects of various external forces on the self-assembly process are worth exploring. For instance, alternating electric or magnetic fields could influence particle orientations and interactions, potentially leading to assembly structures overcoming equilibrium limitations. Stochastic perturbation is another choice of external force that might introduce randomness that can hinder or enhance the assembly process. This exploration will also need to consider how these forces are influenced by key parameters such as system size, patch–patch interactions, and binding affinity between different patchy particles to comprehensively understand their impact on self-assembly. By gaining all these insights, we will extend our approach from simplified models of patchy particles to more complex representations of biological proteins and explore their self-assembly under external influences.

## Supporting Information Available

Detailed sections on the implementation of square wave potential in nonequilibrium MD simulations, comparative dynamics of self-assembly in patchy particle systems, the influence of patch length on self-assembly kinetics and stability, the impact of number density on self-assembly, and the sustained order parameter in nonequilibrium dynamics, featuring: order parameter (*R*) and total entropy production (*S*) dynamics for 8-patchy particles under external drive (Figure S1); order parameter (*R*) and total entropy production (*S*) variations in 13-patchy particles, showing drive and energy effects (Figure S2); total entropy production (*S*) and order parameter (*R*) variations in 8-patchy particles in equilibrium and nonequilibrium for successful realization of the target (Figure S3); *T*_fas_ and *T*_stable_ changes with patch energy and drive in 10-patch systems under different patch lengths (Figure S4); impact of number density on *T*_fas_ and *T*_stable_ in 10-patch systems under different conditions (Figure S5); consistent *R* growth in 8-patch and 10-patch systems across square wave potential amplitudes (Figure S6); average number of bond formation and dissociation events (Figure S7); variation of *N*_B_ as a function of MC steps for 100 particles with 8-ring specification with and without external drive (Figure S8); variation of *N*_B_ and *N*_R_ as a function of MC steps for 100 particles with 4-ring specification with and without external drive (Figure S9); variation of *N*_B_ as a function of MC steps for 100 particles with 8-ring specification in equilibrium (Figure S10) (PDF)
A series of supplementary movies illustrate the dynamic behavior of the systems: Equilibrium MC, 8 particles, 2 state (Movie S1) (MP4)
Equilibrium MC, 10 particles, 2 states (Movie S2) (MP4)
Equilibrium MC, 13 particles, 2 state (Movie S3) (MP4)
Nonequilibrium MC, 8 particles, 2 states (Movie S4) (MP4)
Nonequilibrium MC, 10 particles, 2 states (Movie S5) (MP4)
Nonequilibrium MC, 13 particles, 2 states (Movie S6) (MP4)
Equilibrium MD, 8 particles, 1 state (Movie S7) (MP4)
Equilibrium MD, 10 particles, 1 state (Movie S8) (MP4)
Nonequilibrium MD, 8 particles, 1 state (Movie S9) (MP4)
Nonequilibrium MD, 10 particles, 1 state (Movie S10) (MP4)
Equilibrium MC, 100 particles, 1 state, 8-ring specification (Movie S11) (MP4)
Equilibrium MC, 100 particles, 1 state, 4-ring specification (Movie S12) (MP4)
