# Supplementary material for: Dissipative Self-Assembly of Patchy Particles under Nonequilibrium Drive: A Computational Study
Source: J Chem Theory Comput. 2024 Oct 4;20(20):8844–61. doi: 10.1021/acs.jctc.4c00856 (PMC11500309; doi:10.1021/acs.jctc.4c00856)
Supplement: Supplementary file 1 — ct4c00856_si_001.pdf [file ct4c00856_si_001.pdf]

Supporting Information: Dissipative self-assembly of patchy particles under nonequilibrium drive: a computational study

Shubhadeep Nag[†] and Gili Bisker[*,†,‡,¶,§,‖]

†*Department of Biomedical Engineering, Faculty of Engineering, Tel Aviv University, Tel Aviv 69978, Israel*

‡*The Center for Physics and Chemistry of Living Systems, Tel Aviv University, Tel Aviv 6997801, Israel*

¶*The Center for Nanoscience and Nanotechnology, Tel Aviv University, Tel Aviv 6997801, Israel*

§*The Center for Light-Matter Interaction, Tel Aviv University, Tel Aviv 6997801, Israel*

‖*The Center for Computational Molecular and Materials Science, Tel Aviv University, Tel Aviv 6997801, Israel*

E-mail: bisker@tauex.tau.ac.il

S1 Total Entropy Production Calculation in MC Simulations

The calculation of entropy production in MC simulations involves summing the contributions from each accepted MC move, where each move alters the system's energy. The contribution is calculated by k_B times the log-ratio of the forward and reverse transition probabilities.[1] Let $P_{forward}$ be the probability of moving forward from one state to another, and $P_{reverse}$ be the probability of moving reverse between the same two states.

During an MC step, if a particle transitions from an initial energy (E_{ini}) to a final energy (E_{final}), the energy change ($\delta E = E_{final} - E_{ini}$) determines the transition probabilities. If $\delta E < 0$, indicating a decrease in energy, the forward transition probability, $P_{forward}$, is 1 and the reverse transition probability, $P_{reverse}$, is $e^{+\delta E/k_B T}$, T is the temperature. Conversely, if $\delta E > 0$, signifying an increase in energy, the forward transition probability, $P_{forward}$, is $e^{-\delta E/k_B T}$, and the reverse transition probability, $P_{reverse}$, is 1. Thus, the contribution to entropy production, namely $k_B \ln(\frac{P_{forward}}{reverse})$, in the case of $\delta E > 0$ is $-\frac{\delta E}{T}$, and in the opposite case, $\delta E < 0$, it is $+\frac{\delta E}{T}$. In MC simulations, entropy production accumulates from these probabilistic energy changes and sums to the total entropy change over the course of the simulation. The total entropy production, S is the sum of these contributions. The resulting total entropy production is in the units of the (molar) energy divided by the temperature, kJ / (mol · K).

S2 Self-Assembly Dynamics in 8 and 13 patchy Particle Systems in MC Simulations

We extend the analysis of the order parameter, R, and the total entropy production, S, to systems of 8 and 13 patchy particles, in addition to the 10 patchy particle system presented in the main text.

The evolution of R for a 8 patchy particle system under equilibrium ($\epsilon_{drive} = 0$) and nonequilibrium ($\epsilon_{drive} = 5$ kJ/mol) conditions is depicted in Fig. S1(a) and Fig. S1(b), respectively, for a patchy interaction value $\epsilon_{patch} = 4$ kJ/mol. In the absence of an external drive, R displays stochastic behavior, oscillating between 0 (i.e., no bond formed) and 0.75 (i.e., 6 bonds formed out of the 8 possible bonds), indicating the inability of the system to self-assemble the target structure. With the external drive, R displays a rapid transition from a disordered state to the fully assembled target, corresponding to $R = 1$ at 1.3×10^6 MC steps. The total entropy production, S, without the drive, shows zero entropy production rate (Fig. S1(c)), in contrast to the nonequilibrium case where the value of S rapidly increases (Fig. S1(d)), reflecting the energy dissipation associated with the use of the external drive.

Similarly, for the 13 patchy particle system with $\epsilon_{patch} = 5$ kJ/mol, R fluctuates randomly only between 0.4 and 0.8 through the entire course of the simulation under equilibrium conditions (Fig. S2(a)), where under nonequilibrium conditions with $\epsilon_{drive} = 7$ kJ/mol, R increases in a step-wise manner until it reaches the value of 1 at 3.9×10^6 MC steps for the first time, which represents a fully assembled structure (Fig. S2(b)). Moreover, S displays fluctuations with mean zero graduate, indicating zero entropy production rate at equilibrium (Fig. S2(c)), whereas with the external drive, S rapidly increases (Fig. S2(d)).

These results demonstrate that external driving forces not only facilitate the self-assembly of patchy particles into a predefined structure but also modulate the thermodynamic signatures of the process. This modulation is evident in the transition to an ordered state captured by the increase in R and S. Furthermore, to provide a comparison, the computation of S and R for both equilibrium and nonequilibrium cases, where each realization reaches the target structure, is plotted in Fig. S3.

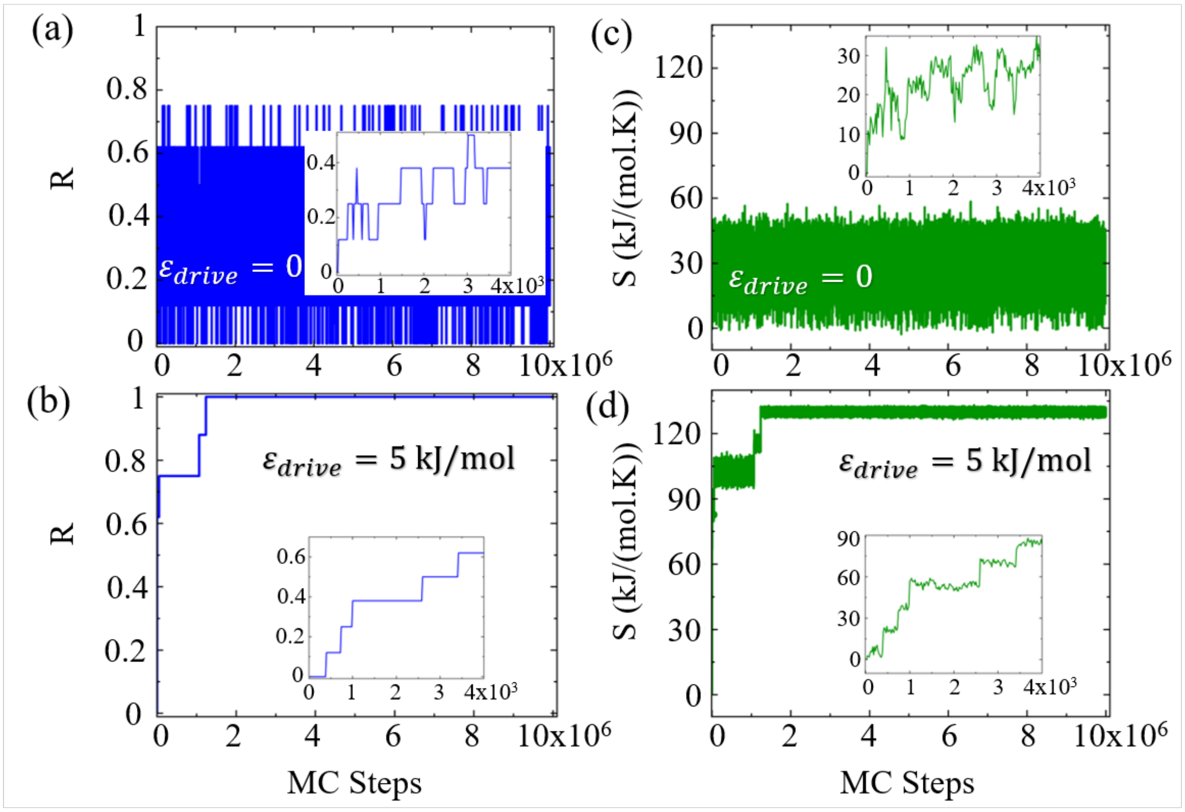

Figure S1: Results of 8 patchy particle system. Order parameter (R) as a function of MC steps (a) in equilibrium and (b) under nonequilibrium conditions with an external drive $\epsilon_{drive} = 5$ kJ/mol. The total entropy production (S) as a function of MC steps (c) in equilibrium and (d) under nonequilibrium conditions with an external drive $\epsilon_{drive} = 5$ kJ/mol. Results are presented for a single MC realization with $\epsilon_{patch} = 4$ kJ/mol. The inset plots show Zoom in into the early simulation steps.

S3 Effect of Patch Length on Self-Assembly Kinetics in MC Simulations

In addition to the the patch length chosen for our main analysis, 0.85 Å, we extend our simulations to include patch lengths of 0.4 Å and 1.1 Å to investigate the effect of this parameter on the results for the 10 patchy particle system. Under equilibrium conditions, both the median self-assembly time, T_{fas} (Fig. S4(a)), and median stability time, T_{stable} (Fig. S4(b)), show a similar trend as a function of the patch interaction energy, ϵ_{patch}, for the 3 patch length values tested.

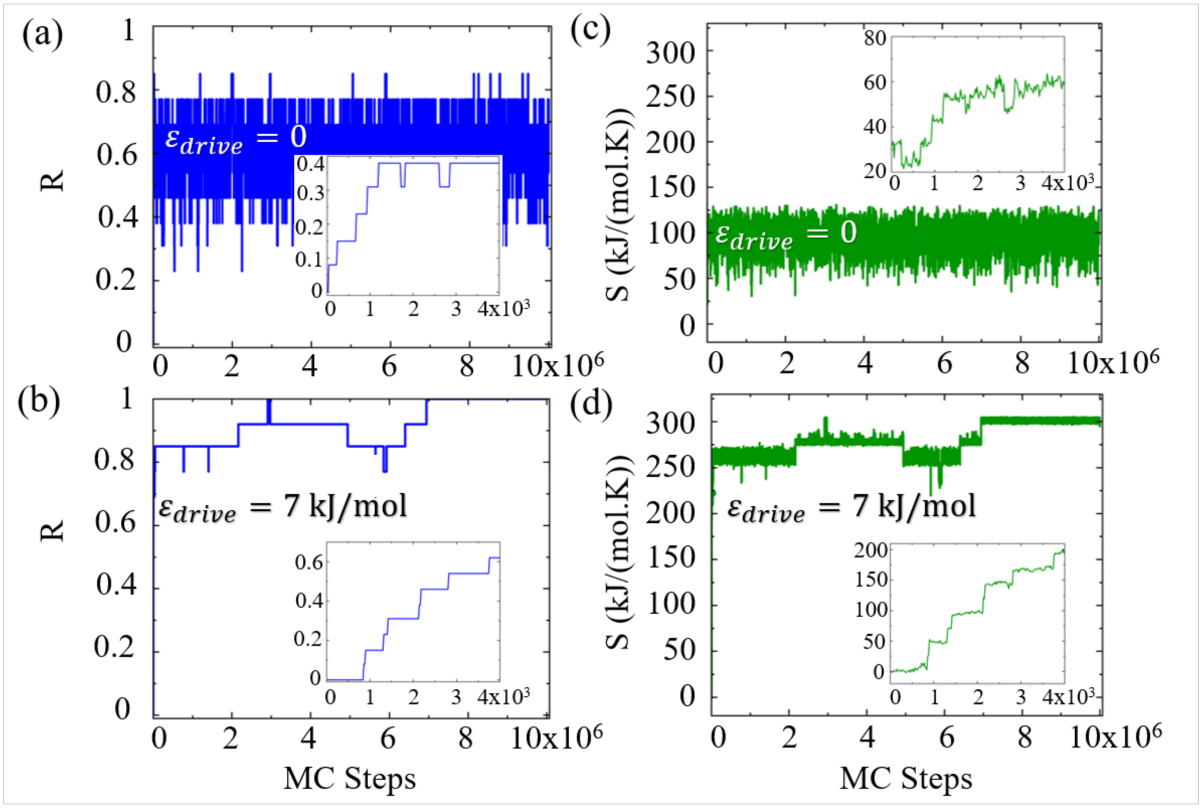

Figure S2: Results of 13 patchy particle system. Order parameter (R) as a function of MC steps (a) in equilibrium and (b) under nonequilibrium conditions with an external drive $\epsilon_{drive} = 7$ kJ/mol. The total entropy production (S) as a function of MC steps (c) in equilibrium and (d) under nonequilibrium conditions with an external drive $\epsilon_{drive} = 7$ kJ/mol. Results are presented for a single MC realization with $\epsilon_{patch} = 5$ kJ/mol.

Under nonequilibrium conditions, with $\epsilon_{patch} = 5$ kJ/mol, we also observe a similar trend of T_{fas} (Fig. S4(c)) and T_{stable} (Fig. S4(d)), showing faster assembly and higher stability, respectively, for increasing drive value ϵ_{drive}, for the various patch lengths. These results confirm that our conclusions hold irrespective of the chosen patch length parameter value.

S4 Effect of Simulation Cell Dimension on Self-Assembly Kinetics in MC Simulations

Understanding the kinetics of self-assembly in the context of number density is important for tuning the properties of assembled structures. In addition to the simulation cell length of

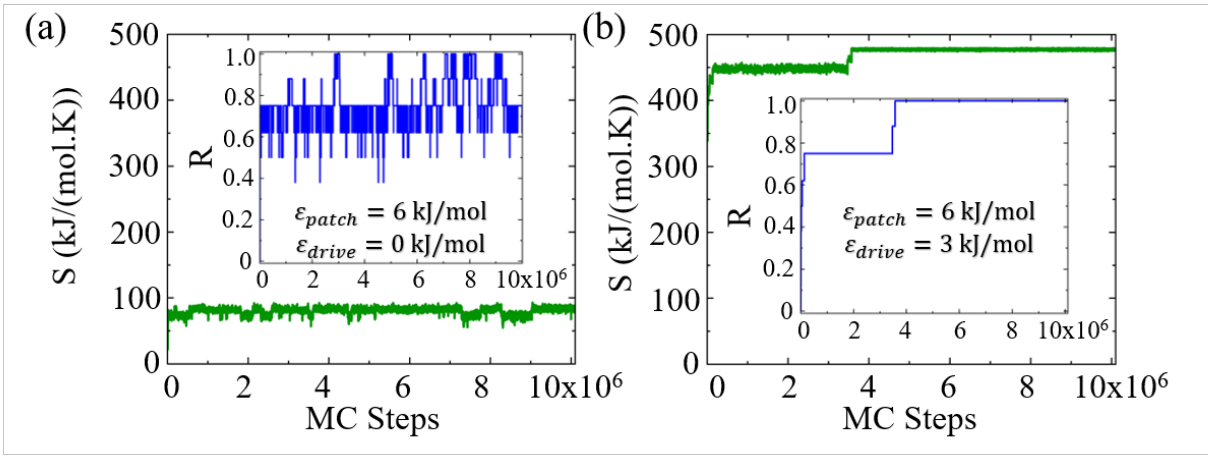

Figure S3: Total entropy production (S) as a function of MC steps (a) in equilibrium and (b) under nonequilibrium conditions with an external drive = 3 kJ/mol. Results are presented for a single MC realization of 8 particle system with ϵ_{patch} = 3 kJ/mol. The insets show the variation of R.

9 Å used for our main analysis, we include additional simulations for a 15 Å and 25 Å cell, for the 10 patchy particle system (Fig. S5). Compared to simulation cells of 9 Å and 15 Å, the values of T_{fas} from both equilibrium and nonequilibrium simulations exhibit higher optimal values in the larger system due to the reduced number density, which leads to less frequent interactions among the patchy particles. Importantly, both T_{fas} and T_{stable} show the same trend in equilibrium and nonequilibrium conditions, further affirming the robustness of our conclusions, which are independent of the simulation cell dimension.

S5 Order Parameter R in Nonequilibrium Molecular Dynamics

The influence of the nonequilibrium driving forces on the self-assembly of patchy particles manifested by the order parameter R is studied for 8 and 10 patchy particle systems in equilibrium and nonequilibrium MD simulations. The average order parameter along a realization serves as an indicator of the structural alignment of the system with the desired target assembly. For the 8 particle (Fig. S6(a)-(c)) and 10 particle (Fig. S6(d)-(f)) systems,

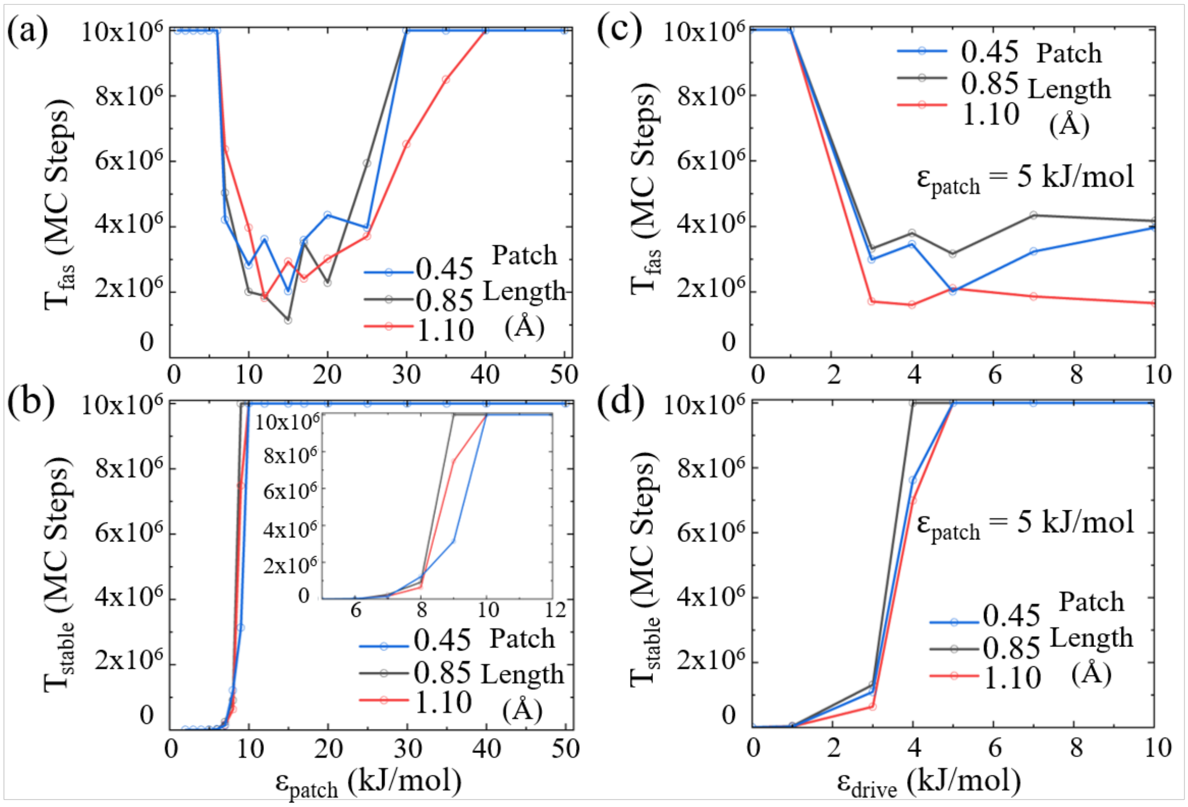

Figure S4: MC simulation results of the 10 patchy particle system from 20 independent realizations for patch lengths of 0.4 Å (blue), 0.85 Å (black), and 1.1 Å (red). (a) Median T_{fas} is plotted for different patchy particle interaction energies, ϵ_{patch}, under equilibrium conditions. (b) Median T_{stable} is plotted for different patchy particle interaction energies, ϵ_{patch}, under equilibrium conditions. Inset: Zoom into the lower ϵ_{patch} range. (c) Median T_{fas} is plotted for $\epsilon_{patch} = 5$ kJ/mol, as a function of the drive value, ϵ_{drive}, under nonequilibrium conditions. (d) Median T_{stable} is plotted for $\epsilon_{patch} = 5$ kJ/mol, as a function of the drive value, ϵ_{drive}, under nonequilibrium conditions.

we see a moderate increase in the average R value with increasing amplitude of the square wave potential, for ϵ_{patch} values of 4.5 kJ/mol, 5 kJ/mol, and 6 kJ/mol, mirroring the increase in target stability observed in Figs. 9.

S6 Bond Statistics from MD Simulation

To investigate the impact of the square wave potential, $U_{square}(t)$, on the assembly kinetics and stability, we track bond formation and dissociation events up to the point of achieving the

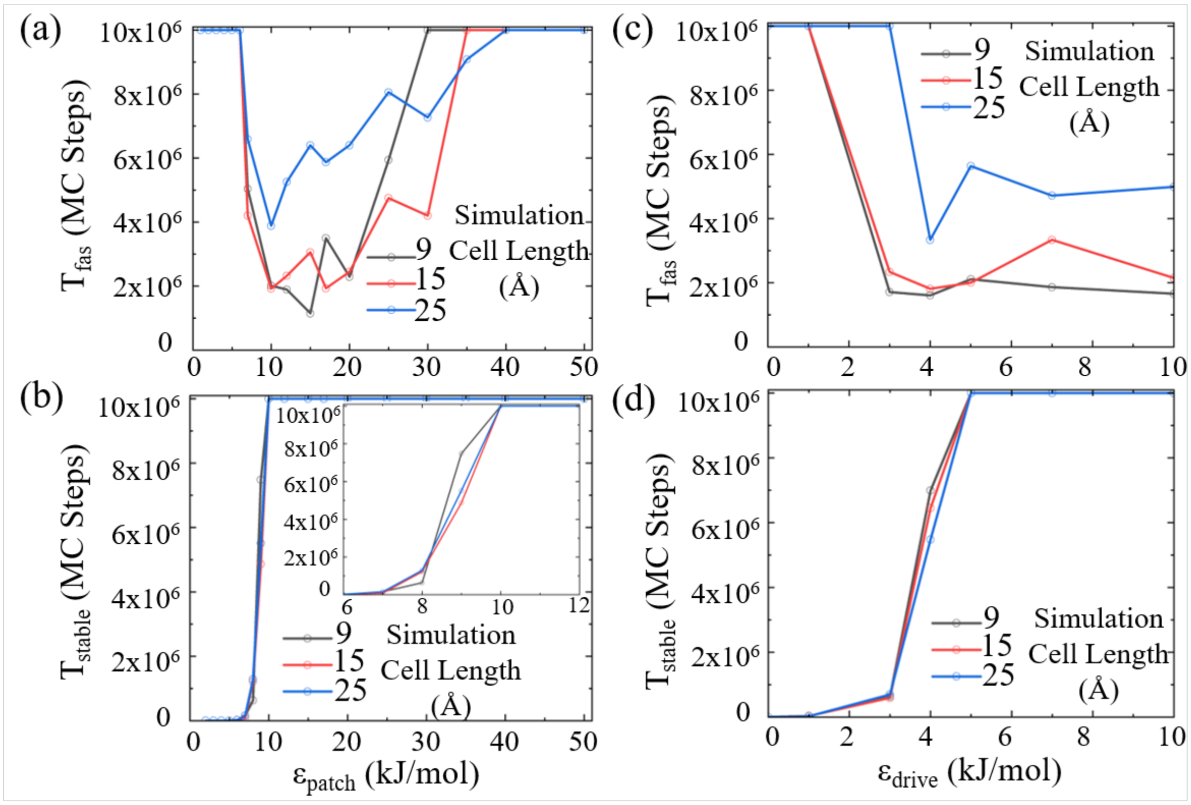

Figure S5: MC simulation results of the 10 patchy particle system from 20 independent realizations for different simulation cell dimensions of 9 Å (black), 15 Å (red), and 25 Å (blue). (a) Median T_{fas} is plotted for different patchy particle interaction energies, ϵ_{patch}, under equilibrium conditions. (b) Median T_{stable} is plotted for different patchy particle interaction energies, ϵ_{patch}, under equilibrium conditions. Inset: Zoom into the lower ϵ_{patch} range. (c) Median T_{fas} is plotted for $\epsilon_{patch} = 5$ kJ/mol, as a function of the drive value, ϵ_{drive}, under nonequilibrium conditions. (d) Median T_{stable} is plotted for $\epsilon_{patch} = 5$ kJ/mol, as a function of the drive value, ϵ_{drive}, under nonequilibrium conditions.

target structure within our nonequilibrium MD simulations, during both the high energy and low energy phases of the square wave potential. Note that for a zero amplitude, the system is effectively at equilibrium, and the bond events are averaged over the entire simulation duration and divided by two, for a fair comparison with the nonequilibrium cases in which the events are counted separately for the two potential values.

Figs. S7(a) and S7(b) present the average number of bond formation events for the 8 particle and 10 particle systems, for a patchy interaction energy of 6 kJ/mol and 5 kJ/mol, respectively. At 0 amplitude, the average number of bonds formed is ~ 3.8 for the 8 patchy

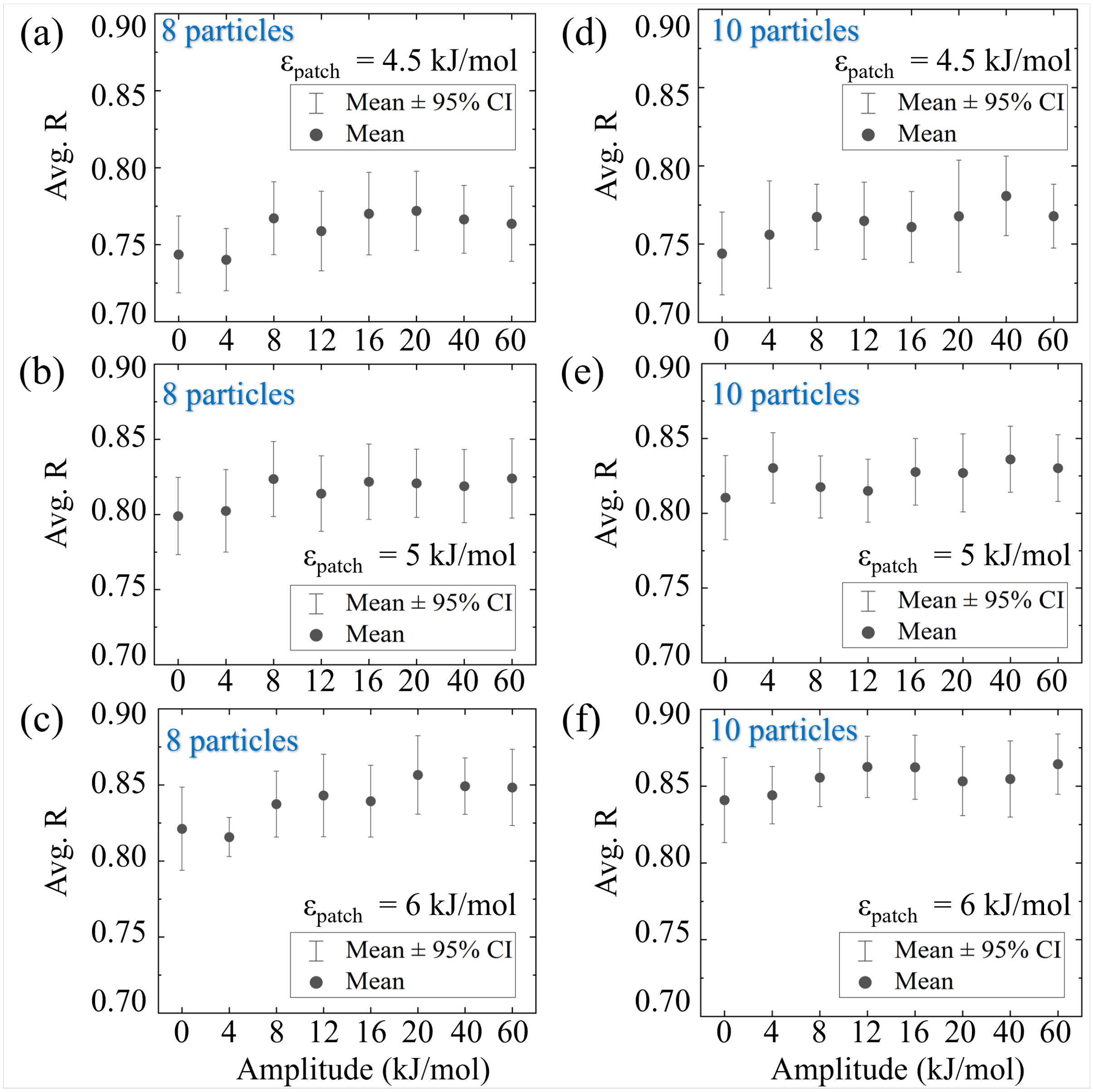

Figure S6: Average order parameter, R, from 20 different MD simulations, as a function of the square wave potential amplitude for 8 particle system and ϵ_{patch} value of (a) 4.5 kJ/mol, (b) 5 kJ/mol, and (c) 6 kJ/mol. Average order parameter, R, from 20 different MD simulations, as a function of the square wave potential amplitude for 10 particle system and ϵ_{patch} value of (d) 4.5 kJ/mol, (e) 5 kJ/mol, and (f) 6 kJ/mol.

particle system and 5.5 for the 10 patchy particle system. As the amplitude increases, the number of bond formations tends to oscillate around this equilibrium value with a significant increase in the standard deviation with respect to equilibrium.

Our analysis indicates that at 0 amplitude, the average number of bonds formed is ~ 3.8

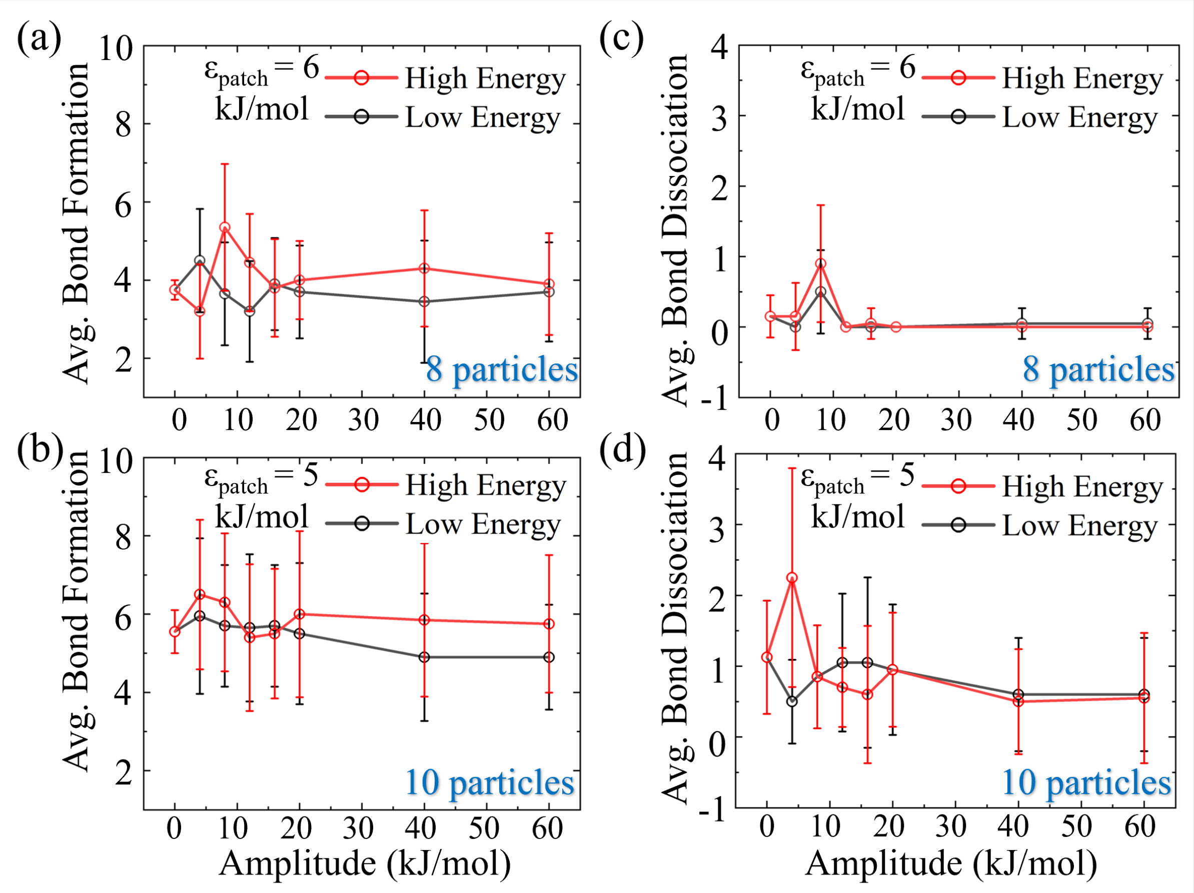

Figure S7: Average number of bond formation events across 20 individual MD simulations during the high energy (red) and low energy (black) phases of the square wave potential, as a function of its amplitude for (a) 8 patchy particles and (b) 10 patchy particles. Similar variations are shown for bond dissociation for (c) 8 patchy particles and (c) 10 patchy particles. The average number of bond dissociation events across 20 individual simulations for (a) 8 patchy particles and (b) 10 patchy particles. Error bars are standard deviations.

for the 8 patchy particle system and 5.5 for the 10 patchy particle system. As the amplitude increases, the number of bond formations tends to oscillate around this equilibrium value with a significant increase in the value of standard deviation with respect to equilibrium. Furthermore, the standard deviation bars for bond formation in the top phase overlap those in the bottom phase, suggesting that while bond formation may appear more frequent in the top phase, the difference is not statistically significant.

In terms of bond dissociation for the 8 patchy particles (Fig. S7(c)), and 10 patchy particles (Fig. S7(d)), the average bond dissociation increases from 0.15 to approximately 0.7

with increasing drive amplitude from 0 up to 8 kJ/mol for 8 patchy particles, whereas for the 10 patchy particle system the average bond dissociation increases from 1.125 to 2.25 for the high energy phase for drive amplitude value of 4 kJ/mol. Beyond these amplitudes, the average bond dissociation reduces to approximately 0 for the 8 patchy particles and approximately 0.55 for the 10 patchy particle system, and the corresponding standard deviations fall below their equilibrium values.

This observation suggests that up to a certain value of drive amplitude, the bond-breaking events increase, whereas, for larger amplitudes, fewer bonds are broken relative to the equilibrium conditions. Therefore, the overall durability of bonds in our patchy particle system increases in response to the increase in the square wave amplitudes, thereby increasing target stability and facilitating target assembly, as seen in Fig. 11 of the main text.

S7 Self-Assembly of Large Systems in the Presence of External Drive

To demonstrate the efficacy of our proposed design principle of employing nonequilibrium force in overcoming equilibrium limitations for larger systems, we present here the equilibrium and nonequilibrium simulations of 100 patchy particles, with patches modeled to form 8 and 4 sided rings. It should be noted that since each particle has two patches and each patch forms one bond, the total number of bonds formed in the system of 100 particles would be 200.

Fig. S8 illustrates the variation of the number of bonds (N_B) as a function of MC steps, highlighting the impact of ϵ_{patch} and ϵ_{drive}. In Fig. S8(a), N_B is plotted for different values of ϵ_{patch} in equilibrium. The results show that higher values of ϵ_{patch} lead to an increased number of bonds, with the number rapidly rising before plateauing at 200. This indicates that stronger interaction energies promote more stable and numerous bond formations. Fig. S8(b) presents N_B for varying ϵ_{drive}. It shows that applying an external drive increases N_B to 200

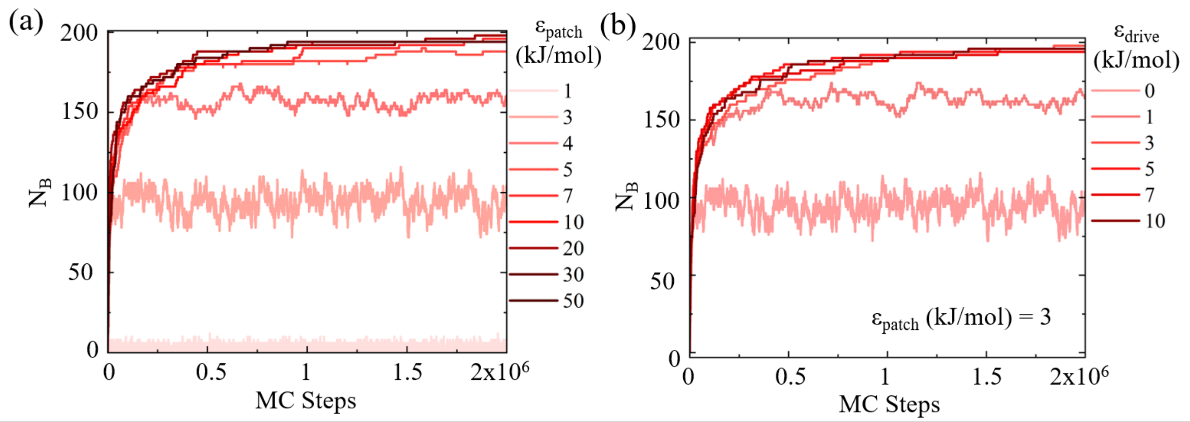

Figure S8: Variation of N_B as a function of MC Steps for (a) different values of interaction energy between patches (ϵ_{patch}) in equilibrium and (b) different values of external drive (ϵ_{drive}) for patchy interaction energy of 3 kJ/mol from single realizations. Two patches are separated from each other by an angular difference of 135^0 (interior angle of the octagon).

while keeping ϵ_{patch} constant at 3 kJ/mol. In equilibrium, the number of bonds formed with the same ϵ_{patch} value (3 kJ/mol) fluctuates around $\sim$ 100. The findings, therefore, reveal that increasing ϵ_{drive} leads to a higher number of bonds for weak ϵ_{patch}, suggesting that the external drive enhances bond formation beyond equilibrium conditions similar to our earlier observation for small systems. In this system, throughout the equilibrium and nonequilibrium conditions, the resultant structure has the shape of a chain with very infrequent formation of large ring structures of $\sim$ 20-sides (see Movie S11).

Fig. S9 illustrates the variation in N_B and the number of ring structures (N_R) as a function of MC steps, with a focus on the impact of the interaction energy between patches and external drives on bond and ring formation. Figs. S9(a) and S9(b) display N_B for different values of interaction energy ϵ_{patch} in equilibrium and different values of ϵ_{drive}, respectively. In Fig. 5, we identified three regions of T_{fas} variation with different ϵ_{patch} values. In the scenario of a large system, T_{fas} represents the MC step when all the particles form bonds with each other. In Figs. S8(a) and S9(a), we observe Regions I and II in a larger system: Region I (low ϵ_{patch}, low N_B) and Region II (increasing ϵ_{patch}, N_B approaching 200). However, Region III is absent in the larger system due to increased particle interactions and mobility, leading to

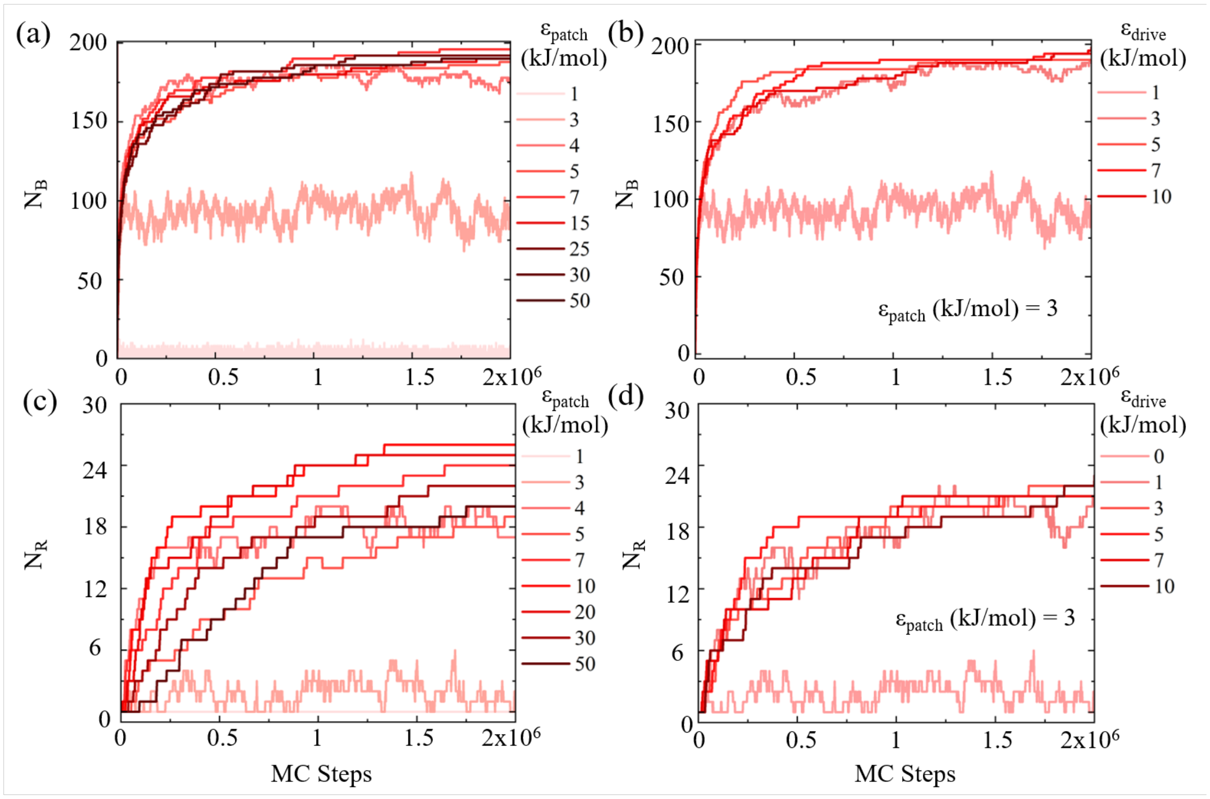

Figure S9: Variation of N_B as a function of MC Steps for (a) different values of interaction energy between patches in equilibrium and (b) different values of external drive for patchy interaction energy of 3 kJ/mol from single realizations. Variation of N_R as a function of MC Steps for (c) different values of interaction energy between patches in equilibrium and (d) different values of external drive for patchy interaction energy of 3 kJ/mol from single realizations. Two patches are separated from each other by an angular difference of 90^0 (interior angle of a square).

diverse structures like rings and chains and preventing a high-energy overbound state. Thus, the larger system only exhibits Regions I and II, reflecting either no structure formation or stable ring and chain formations.

The results are similar to what we observed in Fig. S8. In this system, we observed the formation of a ring structure comprising mainly 4 and 5 particles with the infrequent occurrence of a large polygon of 9 or 10 sides (see Movie S12). Figs. S9(c) and S9(d) present N_R, for varying ϵ_{patch} and then for different ϵ_{drive} for a specific patch interaction energy of 3 kJ/mol, respectively. Here, we observe that an increase in values of ϵ_{patch} resulted in more ring formation (Region II) compared to weak ϵ_{patch} (Region I), with a significant observation

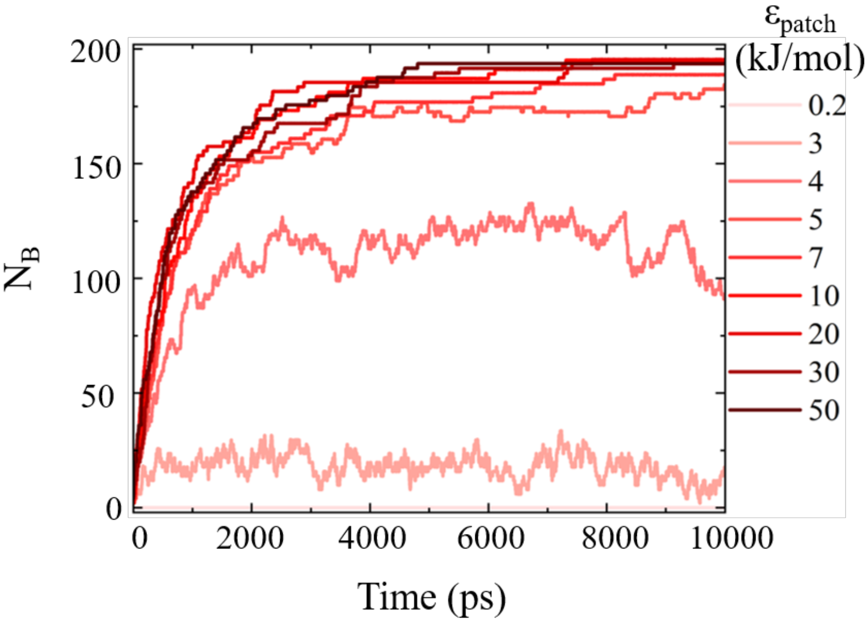

Figure S10: Variation of N_B as a function of time for different values of the interaction energy between patches obtained from equilibrium MD simulations. Two patches are separated from each other by an angular difference of 90^0 (interior angle of a square).

of a lower number of ring formations for higher ϵ_{patch} value akin to region III of Fig. 5 of the main manuscript. Application of ϵ_{drive} results in a higher number of ring formations compared to equilibrium conditions.

Fig. S10 illustrates the variation in N_B as a function of time for different values of ϵ_{patch}, obtained from equilibrium MD simulations. In this case, the patches are separated by an angular difference of 90 degrees (interior angle of a square). The plot shows that for lower ϵ_{patch} values, N_B fluctuates around small values indicating that only a few bonds were formed, where more bonds formed over time with increasing ϵ_{patch} values. Higher values of ϵ_{patch} lead to a greater maximum N_B (200 for the system of 100 particles) and a higher stabilization plateau, indicating more extensive bond formation. Specifically, $\epsilon_{patch} = 50$ kJ/mol results in the highest number of stable bonds, while $\epsilon_{patch} = 0.2$ kJ/mol shows the lowest. This trend is similar to what we observed from Fig. S9(a) for the same system simulated using MC. This result confirms that the observed qualitative behaviors are consistent across both methods and are intrinsic to the assembly process.

S8 Supplementary movies

Movie S1: Equilibrium MC simulation of 8 patchy particles, with 2 internal states, contained within a cubic box of 8 Å side length with reflective boundaries. The simulation employs an interaction energy of 3 kJ/mol for interactions between patches and does not incorporate any external driving forces. Conducted at a temperature of 65 K, the simulation spans 10×10^6 MC steps, with the trajectory recorded every 1000th step.

Movie S2: Equilibrium MC simulation of 10 patchy particles, with 2 internal states, confined within a cubic box of 9 Å in length and featuring reflective boundaries. The simulation utilizes an interaction energy of 3 kJ/mol among the patches, with no external driving forces applied. It is conducted at a temperature of 65 K over 10×10^6 MC steps, with the trajectory being captured every 1000th MC step.

Movie S3: Equilibrium MC simulation of 13 patchy particles with 2 internal states, within a cubic box of 15 Å in length. The simulation is executed with an interaction energy of 4 kJ/mol between the patches in the absence of an external driving force. Conducted at 65 K, the simulation proceeds for 15×10^6 MC steps, with trajectory data recorded every 1000th step.

Movie S4: Nonequilibrium MC simulation of 8 patchy particles, with 2 internal states, contained within a cubic box of 48 Å side length and reflective boundaries. This simulation is executed with an interaction energy of 3 kJ/mol between the patches, under the influence of an external driving force of 7 kJ/mol. Performed at a temperature of 65 K, the simulation extends over 10×10^6 MC steps, with the trajectory being recorded every 1000th step.

Movie S5: Nonequilibrium MC simulation of 10 patchy particles, with 2 internal states, enclosed in a cubic box of 9 Å side length with reflective boundaries. The simulation utilizes an interaction energy of 3 kJ/mol between patches, alongside an external driving force of 7 kJ/mol. Conducted at 65 K, the simulation proceeds for 10×10^6 MC steps, with trajectory data recorded every 1000th step.

Movie S6: Nonequilibrium MC simulation of 13 patchy particles with 2 internal states,

within a cubic box of 15 Å in length. The simulation is executed with an interaction energy of 4 kJ/mol between the patches in the presence of an external driving force of 7 kJ/mol. Conducted at 65 K, the simulation proceeds for 15×10^6 MC steps, with trajectory data recorded every 1000th step.

Movie S7: Equlilbrium MD simulation of 8 patchy particles with 1 internal state within a cubic box of 8 Å in length with periodic boundaries. The simulation is executed with an interaction energy of 4.5 kJ/mol between the patches. It is performed at 40 K for 12000 ps, with the trajectory being recorded every 2 ps.

Movie S8: Equlilbrium MD simulation of 10 patchy particles with 1 internal state within a cubic box of 9 Å in length with periodic boundaries. The simulation is executed with an interaction energy of 5 kJ/mol between the patches. It is performed at 40 K for 12000 ps, with the trajectory being recorded every 2 ps.

Movie S9: Nonequlilbrium MD simulation of 8 patchy particles with 1 internal state within a cubic box of 8 Å in length with periodic boundaries. The simulation is executed with an interaction energy of 4.5 kJ/mol between the patches with a square wave potential with an amplitude of 20 kJ/mol. It is performed at 40 K for 12000 ps, with the trajectory being recorded every 2 ps.

Movie S10: This movie demonstrates a nonequlilbrium MD simulation of 10 patchy particles with 1 internal state within a cubic box of 9 Å in length with periodic boundaries. The simulation is executed with an interaction energy of 5 kJ/mol between the patches with a square wave potential with an amplitude of 12 kJ/mol. It is performed at 40 K for 12000 ps, with the trajectory being recorded every 2 ps.

Movie S11: This movie presents an equilibrium MC simulation of 100 patchy particles with patches of 8-ring specification, with 1 internal state, contained within a cubic box of 30 Å side length with periodic boundaries. The simulation employs an interaction energy of 20 kJ/mol for interactions between patches and does not incorporate any external driving forces. Conducted at a temperature of 40 K, the simulation spans 2×10^6 MC steps, with

the trajectory being recorded every 1000th step.

Movie S12: This movie presents an equilibrium MC simulation of 100 patchy particles with patches of 4-ring specification, with 1 internal state, contained within a cubic box of 30 Å side length with periodic boundaries. The simulation employs an interaction energy of 20 kJ/mol for interactions between patches and does not incorporate any external driving forces. Conducted at a temperature of 40 K, the simulation spans 2×10^6 MC steps, with the trajectory being recorded every 1000th step.

References

(1) Bisker, G.; Polettini, M.; Gingrich, T. R.; Horowitz, J. M. Hierarchical bounds on entropy production inferred from partial information. *Journal of Statistical Mechanics: Theory and Experiment 2017*, 093210.
